# Cardiac Myofibroblast‐Derived Small Extracellular Vesicles Moderate Fibrotic Responses via piRNA‐62788/PIWIL2‐Mediated SRF Silencing

**DOI:** 10.1002/jev2.70204

**Published:** 2025-11-28

**Authors:** Shichao Li, Shuwen Su, Gaopeng Xian, Shunyi Li, Guoheng Zhong, Liming Wen, Dingli Xu, Qingchun Zeng

**Affiliations:** ^1^ State Key Laboratory of Organ Failure Research, Department of Cardiology Nanfang Hospital, Southern Medical University Guangzhou China; ^2^ Guangdong Provincial Key Laboratory of Cardiac Function and Microcirculation Southern Medical University Guangzhou China

**Keywords:** angiotensin II, cardiac remodelling, heart failure, Piwi‐interacting RNA, serum response factor, small extracellular vesicles

## Abstract

During fibrogenesis, certain negative feedback loops are elicited to restrain persistent and hyperactive fibrotic responses. Activated fibroblasts have been found to acquire anti‐fibrotic phenotypes. However, the specific inhibitory modulators remain largely enigmatic. Thus, the present study aimed to examine the intrinsic autoregulatory mechanisms of fibroblasts. Here, we demonstrated that angiotensin II (AngII)‐primed cardiac myofibroblast moderated subsequent profibrotic activation. More importantly, this suppressive action was dependent on small extracellular vesicles (sEVs). Strikingly, small RNA sequencing identified an abundant presence of Piwi‐interacting RNAs (piRNAs) in sEVs. In cultured primary cardiac fibroblasts, piRNA‐62788 was induced by AngII receptor type 2 (AT_2_R) stimulation and encapsulated into sEVs. Furthermore, fibrogenic responses were attenuated by piRNA‐62788 overexpression, whereas aggravated by piRNA‐62788 knockdown. In a mouse model of transverse aortic constriction, either piRNA‐62788 agomir or circulating sEVs of patients with heart failure (HF) mitigated adverse cardiac remodelling, while piRNA‐62788 inhibitor‐containing sEVs accentuated myocardial fibrosis. Mechanistically, piRNA‐62788 formed a functional complex with PIWI‐like protein 2 (PIWIL2) and bound to the 3’ untranslated region (UTR) region of serum response factor (*Srf*) mRNA transcripts, leading to inhibition of the SRF signalling. Additionally, plasma sEV‐derived piRNA‐62788 was significantly upregulated in HF patients and negatively correlated with left ventricular ejection fraction. Collectively, we uncovered a protective negative feedback circuit controlled by AngII/AT_2_R/sEVs axis. Understanding this endogenous anti‐fibrotic pathway may hold therapeutic promise in HF.

## 1 Introduction

Heart failure (HF) is a major cause of morbidity and mortality across the world (Ponikowski et al. 2016). Cardiac fibrosis, a common pathology in multiple cardiovascular conditions, is excessive synthesis and deposition of extracellular matrix proteins in the myocardium, leading to increased myocardial stiffness, reduced ventricular compliance and ultimately cardiac dysfunction (Oatmen et al. 2020). A key cellular event in the pathogenesis of fibrosis is the differentiation of quiescent fibroblasts into contractile myofibroblasts (MyoFbs) (Younesi et al. 2024; Henderson et al. 2020). The myocardin‐related transcription factor (MRTF)/serum response factor (SRF) module plays a dominant role in promoting the migratory and contractile MyoFb phenotype (Lighthouse and Small 2016; Davis and Molkentin 2014).

During fibrogenesis, certain negative feedback loops are elicited to restrain persistent and hyperactive pathologic processes. For example, angiotensin II (AngII) type 1 receptor (AT_1_R) and AT_2_R share a similar affinity for AngII but exert counteracting actions (Gunther 1984). Most of the canonical outcomes of renin‐angiotensin system activation are mediated by AT_1_R, while AT_2_R has anti‐fibrogenic, anti‐proliferative, anti‐inflammatory and anti‐hypertensive effects (Fatima et al. 2021). Like AT_1_R versus AT_2_R, Smad (small mother against decapentaplegic) 6/7 induced by transforming growth factor‐β1 (TGF‐β1) antagonizes Smad2/3 signalling (Humeres et al. 2022, 2024). Surprisingly, disruption of Smad3 leads to more MyoFbs in the infarcted heart (Dobaczewski et al. 2010). MyoFb‐specific Smad3 knockout mice exhibit early systolic dysfunction and accentuated replacement fibrosis (Russo et al. 2019; Weng et al. 2023). Unfortunately, considerably less is known regarding the endogenous anti‐fibrotic signal pathways.

Small extracellular vesicles (sEVs) are cell‐derived membranous structures that originate from the endosomal system (van Niel et al. 2018). A growing body of evidence suggests that sEV‐mediated crosstalk modulates fibrotic responses. For instance, macrophage‐derived sEVs attenuate the progression of pulmonary fibrosis (Guiot et al. 2020). sEVs released from hypoxic cardiomyocytes inhibit fibroblast proliferation and activation (Li et al. 2021). Interleukin‐1β (IL‐1β)‐activated lung fibroblasts produce anti‐fibrotic sEVs (Lacy et al. 2019). sEVs isolated from TGF‐β‐treated lung fibroblasts or patients with idiopathic pulmonary fibrosis are able to downregulate the expression of fibrogenic proteins (Martin‐Medina et al. 2018). However, the existence of analogous anti‐fibrotic signals in heart remains unclear.

P‐element‐induced wimpy testis (Piwi)‐interacting RNAs (piRNAs) are a type of small non‐coding RNAs that are 21–35 nucleotides in length (Ozata et al. 2019). Abundantly and differentially expressed piRNAs are discovered in cardiomyocytes during cardiac hypertrophy (Rajan et al. 2016). DQ726659/PIWI‐like protein 4 (PIWIL4) complex participates in the regulation of cardiac hypertrophy (Gao et al. 2020). piR‐000691 aggravates fibroblast activation by targeting apelin (Chen et al. 2024). Dysregulated serum‐originated sEV‐derived piRNAs are identified in patients with HF (Yang et al. 2018), although their functional relevance to cardiac remodelling has not yet been directly addressed.

A recent study revealed that in the pressure‐overloaded heart, cardiac MyoFbs acquire protective anti‐fibrotic phenotypes (Humeres et al. 2024). In the current study, we hypothesized that activated MyoFb‐derived sEVs may be a critical endogenous negative regulator of fibrogenic responses.

## 2 Materials and Methods

The study was approved by the medical ethics committee of Southern Medical University and was carried out according to the guidelines from Directive 2010/63/EU of the European Parliament on the protection of animals used for scientific purposes (NFYY‐2020‐0837). Human study has been conducted in accordance with the principles of the Declaration of Helsinki, and approved by the medical ethics committee of Southern Medical University (NFEC‐2024‐401). Written informed consent has been obtained from all participants.

### 2.1 Cell Culture

Primary cardiac fibroblasts or cardiomyocytes were separated and cultured from newborn Sprague‐Dawley rats or C57BL/6 mice by enzymatic digestion as described previously (Lyu et al. 2015; Bang et al. 2014). Briefly, the hearts from 1‐ to 3‐day‐old neonatal rats or mice were removed, finely mince and digested with trypsin plus type II collagenase under constant stirring at 37°C. Several digestion steps were repeated before the collected primary cells (fibroblasts and cardiomyocytes) were passed through a cell strainer (75 µm) and centrifuged at 120 *g* for 10 min. The cells were then seeded onto culture dishes and allowed to attach for 90 min. The unadherent cells (cardiomyocytes) were collected and maintained in DMEM/F12 containing 15% foetal bovine serum (FBS), 1% penicillin/streptomycin and 5‐bromo‐2′‐deoxyuridine (BrdU) (0.1 mM) at 37°C in 5% CO_2_. The attached cardiac fibroblasts were plated onto gelatine‐coated plastic culture dishes in DMEM/F12 supplemented with 10% FBS and 1% penicillin/streptomycin at 37°C in 5% CO_2_. When grown to 70%–80% confluence, the cells were gently digested with 0.125% trypsin plus EDTA (Gibco, Cat#: 25200072) and passaged at 1:3. Cardiac fibroblasts were used between passages 1 and 3.

Adult C57BL/6 mice were euthanized. Hearts were removed, washed with D‐Hank's solution, cut into pieces and digested at 37°C for 1 h with 2.5 mg/mL Liberase TL (Roche, Cat#: 5401020001). Then, the cells were collected for culture following the same procedures described above.

Primary human cardiac fibroblasts were purchased from BIOESN (Shanghai, China) (Cat#: BES2579HC) and cultured as indicated by the manufacturer.

### 2.2 Direct Contact Co‐Culture Assay

Cardiac fibroblasts were untreated or stimulated with angiotensin II (AngII) (100 nM) (Sigma, Cat#: A9525) for 24 h. Subsequently, untreated fibroblasts (Fbs) and AngII‐activated myofibroblast (MyoFbs) were trypsinised, counted and mixed at a ratio of 1:1 to performed direct contact co‐cultures of Fb plus Fb, Fb plus MyoFb, or MyoFb plus MyoFb. The co‐cultures were then treated with or without transforming growth factor‐β1 (TGF‐β1) (10 ng/mL) (PeproTech, Cat#: 100‐21) for 72 h.

### 2.3 Conditioned Medium Preparation

Cardiac fibroblasts were untreated or treated with AngII (100 nM) for 24 h. The cells were then washed with PBS and given fresh DMEM/F12 containing 1% sEV‐depleted FBS (contaminating bovine sEVs were depleted by ultracentrifugation at 110,000 *g* for 16 h at 4°C) to generate conditioned medium. After 72 h, the supernatants were collected and pre‐cleared by centrifugation at 300 *g* for 10 min to eliminate floating dead cells. For some experiments, the conditioned medium was ultracentrifuged at 110,000 *g* for 70 min, and pellets (sEVs) were discarded. The remaining supernatants were referred as to sEV‐depleted conditioned medium.

### 2.4 sEV Isolation and Purification

Cardiac fibroblasts were grown to 70%–80% confluency. The cells were then washed with PBS, and serum‐starved (0.1% sEV‐depleted FBS) overnight before they were either untreated or stimulated with AngII (100 nM) in fresh DMEM/F12 supplemented with 1% sEV‐depleted FBS. Following 72 h of culture, conditioned medium of untreated fibroblasts and AngII‐activated myofibroblasts were collected, and the sEVs were isolated and purified by several centrifugation and filtration steps as described previously (Witwer et al. 2013; Hill et al. 2013; Théry et al. 2006). In brief, the conditioned medium was successively centrifuged at 300 *g* for 10 min, 2000 *g* for 10 min and 10,000 *g* for 30 min, and then filtrated through 0.22 µm filter. Subsequently, the supernatant was subjected to ultracentrifugation at 110,000 *g* for 70 min, followed by an additional washing step of the sEV‐containing pellets with PBS at 110,000 *g* for 70 min (ultracentrifuge: Beckman Coulter, L‐80XP; rotor type: swinging‐bucket, SW32Ti). All centrifugations were performed at 4°C. sEV‐containing pellets were resuspended in 100–200 µL PBS and stored at −80°C. sEVs were quantified by measuring protein content using Micro BCA assay kit (Thermo Scientific, Cat#: 23235). For in vitro treatment, 10 µg (approximately 1 × 10^10^ particles) of sEVs on the basis of protein measurement were added to 1 × 10^6^ recipient cells.

For isolation of sEVs from human plasma, blood samples (5–10 mL) were obtained using vacutainer with anticoagulant sodium citrate between 6:00 AM and 8:00 AM. Blood samples were incubated for 30 min at room temperature, and then centrifuged at 3000 rpm for 15 min. The upper layer containing plasma was then collected and subjected to the same multi‐step centrifugation protocol described above.

### 2.5 Transmission Electron Microscopy

sEV pellets were resuspended in 2% PFA and loaded onto formvar carbon‐coated copper grids at 40°C for 20 min, followed by washing with PBS. sEV pellets were post‐fixed in 1% glutaraldehyde for 5 min prior to rinse with distilled water. The sample grids were contrasted in 4% uranyl‐oxalate solution for 5 min, and subsequently embedded in a mixture of uranyl acetate plus 2% methyl cellulose for 10 min on ice. After removal of excess fluid by filter paper, the sample grids dried for 10 min at room temperature and were stored in a box before they were viewed using a transmission electron microscope.

### 2.6 Nano‐Flow Cytometry

sEV pellets were resuspended in PBS and detected by a Flow NanoAnalyzer (Apogee, Cat#: A50 micro plus) as described previously (Liu et al. 2022). Standard polystyrene and silicon dioxide nanospheres mixture (Apogee, Cat#: 1527) was employed and served as a size reference. Data and images were analysed and processed by Apogee Histogram.

### 2.7 sEV Uptake Assay and Quantitation

sEVs were labelled with a PKH26 or PKH67 Fluorescent Cell Linker Kit (Sigma, MINI26‐1KT; Cat#: MINI67‐1KT) according to the manufacturer's protocol with slight modifications. In brief, PKH dye was added to Diluent C and incubated with sEVs for 5 min at 37°C. Bovine serum albumin (BSA) (1% in PBS) was then added to the solution to bind excess unbound dye. The mixture was further ultracentrifuged at 110,000 *g* for 70 min, and the PKH‐labelled sEV pellets were resuspended in 100 µL PBS. Cells were incubated with PKH‐labelled sEVs at 37°C in 5% CO_2_ for 16 h. For sEV tracing, the cells were then washed, fixed, permeabilized, stained for vimentin and visualized using a confocal microscope. For quantitation of sEV uptake, internalization of PKH‐labelled sEVs was determined by flow cytometry, and quantified based on their mean fluorescence intensity or the percentage of PKH‐positive cells. Channels: PKH26 (excitation = 551 nm, emission = 567 nm); PKH67 (excitation = 490 nm, emission = 502 nm).

### 2.8 Immunofluorescence Staining

Cells or heart frozen sections were initially fixed with 4% paraformaldehyde (PFA). Samples were then permeabilized with 0.3% Triton X‐100 for 10 min. After blocking with 5% normal goat serum for 1 h at room temperature, the samples were incubated with primary antibodies at 4°C overnight. Then, the samples were washed with PBS several times and incubated with appropriate fluorescent dye conjugated secondary antibodies for 1 h at room temperature. The samples were washed with PBS and embedded with mounting medium containing DAPI. The specific primary antibodies are summarized in Table S1.

### 2.9 Western Blot Analysis

Whole cell lysates, tissues and sEVs were extracted using RIPA lysis buffer supplemented with 1% (v/v) protease inhibitor cocktail (Sigma, Cat#: P8340), 2% (v/v) phosphatase inhibitor (Sigma, Cat#: P5726), and 1% (v/v) phenylmethyl sulfonyl fluoride (PMSF). Protein samples were mixed with loading buffer (Bio‐Rad, Cat#: 1610747) plus β‐mercaptoethanol (Sigma, Cat#: M3148), and separated by denaturing SDS‐PAGE, followed by transferring onto a PVDF membrane (Millipore). After blocking with 5% non‐fat milk or BSA, the membrane was incubated with primary antibodies at 4°C overnight. Membrane was incubated with the secondary antibodies conjugated to horseradish peroxidase for 1 h at room temperature before being visualized by enhanced chemiluminescence. The specific primary antibodies are to be found in Table S1.

### 2.10 Scratch Assay

Cardiac fibroblasts were cultivated in 6‐well plates and grown to a confluent monolayer. A uniform scratch through cells was made in the middle of each well using a 200 µL yellow pipette tip. Then, the culture medium was changed to DMEM/F12 containing 1% FBS plus BrdU (0.1 mM). Images were acquired immediately (0 h) and 24 h later. Scratch closure was measured based on the migration distance.

### 2.11 Collagen Pad Contraction Assay

Collagen matrix was prepared by transferring ice‐cold neutralization solution into rat tail collagen stock solution (3.5–4.5 mg/mL) (Advanced BioMatrix, Cat#: 5153). The cells were mixed with collagen working solution (final concentration ≈ 2 mg/mL). Then, the collagen mixture containing cells was dispensed to a plate and incubated at 37°C for 1 h. After gel formation, fresh DMEM/F12 culture medium containing 1% FBS plus BrdU (0.1 mM) was added, and the collagen gel was released from the bottom of the well. Photographs taken every day and the diameter of the gel was determined.

### 2.12 Mitochondrial Membrane Potential (MMP) Assay

MMP was measured using a JC‐10 assay kit (Sigma, Cat#: MAK160). Briefly, cardiac fibroblasts were loaded with JC‐10 dye and incubated at 37°C for 1 h. Red‐fluorescent aggregates and green‐fluorescent monomers were monitored by microscope.

### 2.13 Annexin V/Propidium Iodide (PI) Apoptosis Assay

The percentage of cells undergoing apoptosis was determined using a Cell Apoptosis Kit (Invitrogen, Cat#: V13241) per manufacturer's protocol. In brief, cardiac fibroblasts were pre‐treated as indicated before harvest. After washing with cold PBS, the cell suspension was stained with annexin V plus PI, and determined using flow cytometry.

### 2.14 Small RNA Sequencing

Small RNA sequencing was conducted as a technical service at RiboBio (Guangzhou, China). Total RNA was extracted from neonatal rat cardiac fibroblast‐derived sEVs. Small RNA fraction was enriched using native polyacrylamide gel electrophoresis. Purified small RNA fragments were ligated with both 3′ and 5′ adaptors, followed by being reverse transcribed and amplified. The sequencing was performed on HiSeq 2500 (Illumina). MiRNA sequences were annotated according to miRBase. Database of piRBase was used to identify piRNA by BLAST as described previously (Huang et al. 2010). Other small RNAs were annotated by Rfam database.

### 2.15 Real‐Time Quantitative Reverse Transcription (qRT)‐PCR

Total RNA was extracted from samples using TRIzol reagent (Invitrogen, Cat#: 15596) and purified using Total RNA kit II (Omega Bio‐tek, Cat#: R6934) or miRNA kit (Omega Bio‐tek, Cat#: R6842) according to the manufacturer's instructions. For mRNA, reverse transcription was performed using revertAid First Strand cDNA Synthesis Kit (Thermo Scientific, Cat#: K1622). For miRNA or piRNA, a poly‐A tail was initially added to the 3′ end by poly‐A polymerase, and the reverse transcription reactions were then performed using an oligo‐dT adaptor primer (Accurate Biology, Cat#: AG11716). The resulting cDNA served as a template for real‐time PCR, and mixed with SYBR Green Master Mix (Applied Biosystems, Cat#: A25742) and designated primer sets. *Gapdh* (for mRNA) or *U6* (for miRNA and piRNA) was used as endogenous control. Further details about primer sequences for specific gene assays are shown in Table S2.

### 2.16 Droplet Digital PCR

Absolute copy number of sEV‐derived small RNA (miRNA and piRNA) was measured using droplet digital PCR. PCR reaction mixture (Bio‐Rad, Cat#: 186‐4036) was combined with droplet generation oil (Bio‐Rad, Cat#: 1863004) in droplet generator (Bio‐Rad, Cat#: 1864002), and the water‐in‐oil droplets were then transferred into a 96‐well plate to performed the PCR amplification per the manufacturer's recommendations. Droplet reader (Bio‐Rad, Cat#: 1864003) was utilized to evaluate the small RNA copy, and the results were analysed using QuantaSoft software.

### 2.17 Cell Transfection

For RNA interference, and piRNA overexpression/inhibition in vitro, transfection was performed by a liposomal‐based method using Lipofectamine 3000 (Invitrogen, Cat#: L3000015) according to the manufacturer's protocol. Full details about the sequences can be found in Table S3.

### 2.18 RNA Immunoprecipitation (RIP) Assay

A commercial kit (Geneseed, Cat#: P0101) was used according to the protocol provided by the manufacturer. In brief, cell lysates were incubated with protein A/G magnetic beads coated with control IgG or anti‐PIWIL2 antibody at 4°C overnight, followed by rinse and elution. Subsequently, immunoprecipitated RNA was purified and subjected to qRT‐PCR analysis to determine the enrichment of piR‐62788 or serum response factor (*Srf*) mRNA in the PIWIL2 fraction.

### 2.19 RNA Decay Assay

To determine the stability of *Srf* mRNA, actinomycin D (5 µg/mL) was used to suppress transcription. Cardiac fibroblasts were harvested for measuring the respective *Srf* mRNA levels at the indicated time points. Half‐life (t_1/2_) was calculated by degradation rate.

### 2.20 Dual‐Luciferase Reporter Assay

The 500 bp fragments of the 3′ untranslated regions (UTR) region of *Srf* encoding gene containing the potential piR‐62788 binding sites were cloned into psiCHECK2 vector. The same gene fragments, served as a control, were mutated to mute the seed sequence and then cloned into vector. The putative complementary sequences were as follows:

Wildtype (WT): 5′‐TCTCCTGGGATGTCGACCCAGGA‐3′

Mutant (MUT): 5′‐TCTCCTGGGATGTCGTGGGTCCT‐3′

The resulting construct was co‐transfected with piR‐62788 mimic or negative control into HEK293T cells using Lipofectamine 2000. After 48 h, luciferase activities were detected using Double‐Luciferase Reporter Assay Kit (TransGen, Cat#: FR201) according to the manufacturer's protocol.

### 2.21 Animal Experiments

Male 8‐week‐old (weight, 18–22 g) C57BL/6 mice were subjected to transverse aortic constriction (TAC) surgery to establish left ventricular pressure overload‐induced chronic HF model as described previously (Bang et al. 2014). Mice were anesthetized with sodium pentobarbital (50 mg/kg intraperitoneally) and ventilated with a rodent respirator. All mice were randomly assigned to the following groups: (1) Sham; (2) TAC; (3) TAC + agomirNC, mice were treated immediately after surgery with a tail vein injection of agomir negative control every week; (4) TAC + agomir, mice were treated immediately after surgery with a tail vein injection of piR‐62788 agomir (2.5 nmol) every week.

For some experiments, piR‐62788 inhibitor or scrambled negative control was transfected into sEVs utilizing electroporation. Briefly, sEVs were mixed with inhibitor/inhibitorNC at a ratio of 1:1 (w/w). This mixture was transferred into an ice‐cold 4 mm cuvette and electroporated for 10 pulses at a perforation voltage of 110 V and capacitance of 940 µF (CUY21 EDIT II, BEX, Japan). Mice were intramyocardially injected at three sites in the left ventricle at a dose of 3 × 10^9^ particles per animal during TAC surgery. After 4 weeks, the echocardiographic images were obtained before the hearts were harvested.

### 2.22 Echocardiography

Mice were anesthetized by 2% inhaled isoflurane. Two‐dimensional parasternal images and M‐mode tracings were recorded both on the long‐ and short‐axis at the level of papillary muscle to determine the cardiac function and structure using a Vevo 2100 echocardiography system. Parameters of left ventricular internal dimension at end‐diastole (LVIDd), left ventricular internal dimension at end‐systole (LVIDs), left ventricular ejection fraction (LVEF) and fractional shortening (FS) were measured or calculated. The operator was blinded to the group allocation.

### 2.23 Histology for Hypertrophy and Fibrosis

Mice were euthanized by an overdose of sodium pentobarbital (150 mg/kg intraperitoneally). The hearts were harvested and fixed with 4% PFA for 24 h before they were dehydrated and embedded in paraffin. Serial sections of 4–6 µm were prepared, dried, deparaffinized and washed. Cardiomyocyte diameter was detected using wheat germ agglutinin (WGA) staining (10 µg/mL) (AAT Bioquest, Cat#: 25559). Myocardial fibrosis was stained using Masson's Trichrome kit (MXB Biotechnologies, Cat#: MST‐8003) and PicroSirius red kit (Solarbio, Cat#: G1472) according to the manufacturer's protocol.

### 2.24 Enzyme‐Linked Immunosorbent Assay (ELISA)

Mouse plasma concentrations of N‐terminal pro‐brain natriuretic peptide (NT‐proBNP) were measured using a commercially available ELISA kit (Meimian, Cat#: MM‐0558M2) according to the manufacturer's protocol. The limit of detection is 1.0 pg/mL, with an assay range of 37.5–1200 pg/mL.

### 2.25 Patients and Clinical Specimens

For collection of peripheral blood, HF was diagnosed according to relevant guidelines (Ponikowski et al. 2016), and healthy individuals with normal cardiac function were recruited from participants in annual health examinations. Thirteen patients and thirteen healthy controls were enrolled in the present study (Table S4). For collection of heart tissue samples, atrial appendage specimens were obtained from a patient who underwent heart surgery for valve heart disease and atrial fibrillation, and a healthy individual who died of motor vehicle accident.

### 2.26 Statistical Analysis

Data are presented as mean ± SEM. All statistical analyses were carried out using Graphpad Prism software. A *p* value < 0.05 was considered statistically significant. Two‐tailed student's *t*‐test or one‐way ANOVA followed by Tukey's post hoc test, was utilized to compare the data between two groups or among multiple groups, respectively.

## 3 Results

### 3.1 AngII‐Primed MyoFbs Produce Anti‐Fibrotic Mediators in a Negative Feedback Mode

**FIGURE 1:**
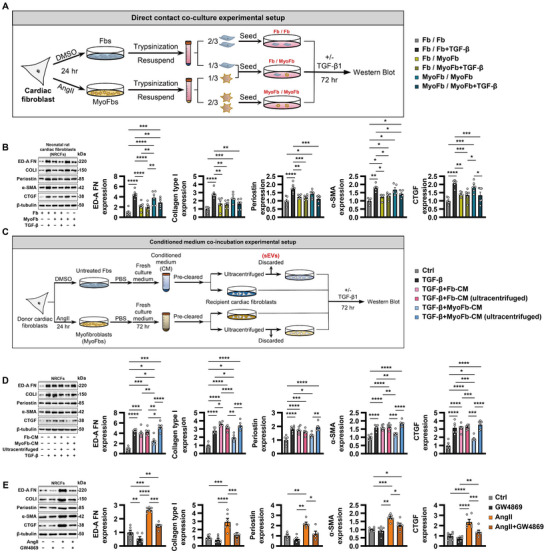
**AngII‐primed MyoFbs produce anti‐fibrotic mediators in a negative feedback mode. (A and B)** Direct contact co‐culture assay. Expression of profibrogenic proteins (alpha smooth muscle actin [α‐SMA], collagen type I [COLI], connective tissue growth factor [CTGF], fibronectin ED‐A domain [ED‐A FN] and periostin) was determined by Western blot. **(C and D)** Conditioned medium (CM) co‐incubation. Cardiac myofibroblasts were induced by angiotensin II (AngII). CM was collected and pre‐cleared. Ultracentrifugation was used to deplete sEVs from CM, and the remaining supernatants were referred as to sEV‐depleted CM. **(E)** Effect of exosomal inhibitor GW4869 (10 µM) on fibroblast activation. Data shown are mean ± SEM. * *p* < 0.05, ** *p* < 0.01, *** *p* < 0.001, **** *p* < 0.0001. *n* = 5–7, each dot represents a biologically independent sample. Significance was analysed by one‐way ANOVA followed by Tukey's post hoc test. DMSO, dimethyl sulfoxide; Fbs, fibroblasts; MyoFbs, myofibroblasts.

To determine the intrinsic autoregulatory capacity of cardiac fibroblasts, we performed a direct contact co‐culture assay (Figure 1A). When either half of the co‐culture was pre‐treated with AngII prior to establishing the co‐culture, TGF‐β1‐induced expression of fibrogenic proteins was robustly inhibited compared to co‐cultures in which neither source population was pre‐treated with AngII (Figure 1B).

Next, conditioned medium co‐incubation assay was used to confirmed the paracrine mediators released by MyoFbs (Figure 1C). Conditioned medium of AngII‐activated MyoFbs, but not untreated fibroblasts, significantly blunted the expression of fibrosis‐associated proteins in response to TGF‐β1 (Figure 1D). Furthermore, depletion of sEVs by ultracentrifugation completely abolished these suppressive actions of conditioned medium.

In addition, we also found that pharmacological inhibition of the formation and secretion of sEVs by GW4869 repressed the expression of several ECM proteins and fibrogenic mediators, especially in the presence of AngII treatment (Figure 1E), highlighting the sequestration of anti‐fibrotic molecules within cells. These data suggested that sEVs may serve as a key regulator of phenotype switch during the progression and regression of fibrosis.

### 3.2 MyoFb‐Derived sEVs Are Able to Suppress Profibrotic Activation

**FIGURE 2:**
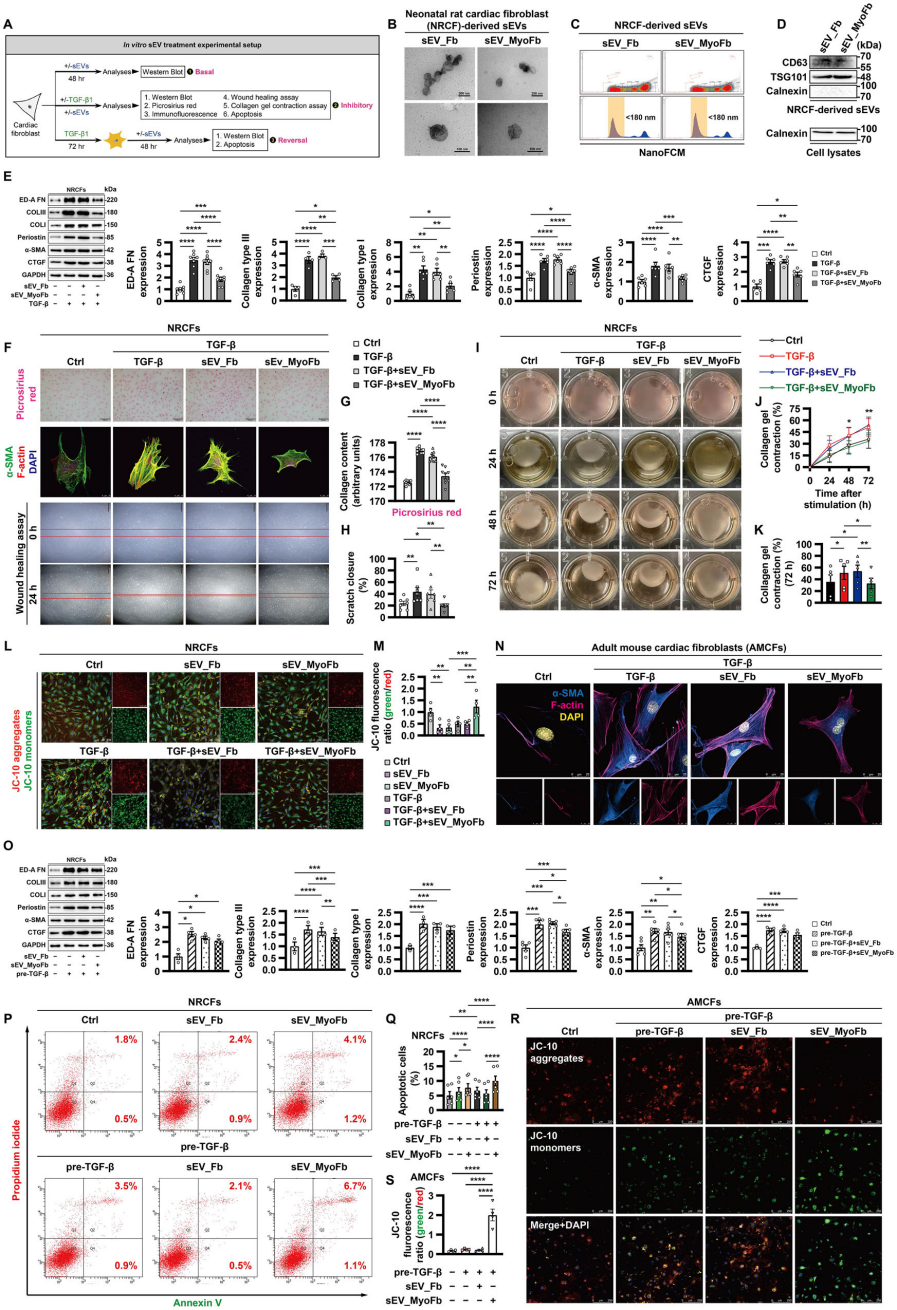
**MyoFb‐derived sEVs are able to suppress profibrotic activation. (A)** Experimental setup. Small extracellular vesicles (sEVs) were isolated from conditioned medium of untreated fibroblasts (Fbs) and angiotensin II‐activated myofibroblasts (MyoFbs) by ultracentrifugation. **(B–D)** sEV characterization was confirmed by transmission electron microscopic analysis (B), nano‐flow cytometry (NanoFCM) (C), and Western blot (D). **(E–H)** NRCFs were co‐cultured with sEVs (10 µg/mL) for 6 h, followed by exposure to TGF‐β1 (10 ng/mL) for 72 h. Expression of fibrogenic proteins was determined by Western blot (E). The cells were stained with picrosirius red; or α‐smooth muscle actin (SMA) (green), F‐actin (red) and nuclei (DAPI, blue) (F). Quantification of picrosirius red staining (G) (*n* = 3 biologically independent samples/nine different fields in each group). Representative photographs of scratch wounds at 0 and 24 h after wounds were made (F), and quantification of migration distances is shown in (H). **(I–K)** Collagen pad contraction assay. NRCFs were co‐cultured with sEVs (10 µg/mL). After 6 h, the cells were removed by trypsinization and counted. An equal number of cells were mixed with rat tail collagen solution, and then the mixture was transferred to culture plates. Following polymerization, the collagen gel was allowed to float in fresh culture medium supplemented with or without TGF‐β1 (10 ng/mL). **(L and M)** NRCFs were co‐cultured with sEVs (10 µg/mL) for 6 h, followed by exposure to TGF‐β1 (10 ng/mL) for 24 h. After loading with JC‐10 dye (a mitochondrial membrane potential probe), images were acquired using microscope and the mean fluorescence intensity ratio of monomers (green) to aggregates (red) was calculated. **(N)** Validation in adult mouse cardiac fibroblasts (AMCFs). **(O–Q)** NRCFs were pre‐treated with TGF‐β1 (10 ng/mL) for 72 h. Then, the cells were co‐cultured with sEVs (10 µg/mL) for 48 h. Expression of fibrogenic proteins was determined by Western blot (O). Annexin V‐positive cells were quantified by flow cytometry (P and Q). **(R and S)** Validation in AMCFs. Data shown are mean ± SEM. * *p* < 0.05, ** *p* < 0.01, *** *p* < 0.001, **** *p* < 0.0001. *n* = 3–8, each dot represents a biologically independent sample except (G). Significance was analysed by one‐way ANOVA followed by Tukey's post hoc test. TGF‐β, transforming growth factor‐β; TSG101, tumour susceptibility gene 101 protein; COL, collagen; CTGF, connective tissue growth factor; ED‐A FN, fibronectin ED‐A domain.

In order to thoroughly elucidate the role of MyoFb‐derived sEVs in fibrotic responses, we performed in vitro sEV treatment as depicted in Figure 2A. Extracellular vesicles isolated from conditioned medium were characterized extensively. Transmission electron microscopy and nano‐flow cytometry indicated characteristic cup‐shaped membrane vesicles with a typical size of 50–150 nm (Figure 2B,C). Western blot showed the presence of signature sEV marker CD63 and tumour susceptibility gene 101 protein (TSG101), whereas calnexin was not detectable (Figure 2D). PKH67‐labelled sEVs were readily internalized by cardiac fibroblasts (Figure S1A). The uptake efficiency of untreated fibroblast‐derived sEVs (sEV_Fb) was slightly higher than that of MyoFb‐derived sEVs (sEV_MyoFb) (Figure S1B–G). Moreover, cardiac fibroblast‐derived sEVs were uptaken most efficiently by cardiac fibroblasts as compared to cardiomyocytes and smooth muscle cells (Figure S1H–J).

Under basal conditions, quiescent phenotype of fibroblasts was hardly affected by sEVs (Figure S2). Interestingly, sEV_MyoFb markedly restricted the expression of fibrogenic proteins, extracellular matrix synthesis, formation of α‐SMA‐positive stress fibres, migration, collagen gel contraction and survival in fibroblasts in response to TGF‐β1 (Figure 2E–N). Furthermore, sEV_MyoFb had modest effects on the expression of fibrogenic proteins in TGF‐β1‐pre‐treated fibroblasts, along with an increased cell death (Figure 2O–S). Collectively, these results pointed to the anti‐fibrotic properties of MyoFb‐derived sEVs.

### 3.3 piR‐62788 Is the Active Element Responsible for the Anti‐Fibrotic Effects of MyoFb‐Derived sEVs

**FIGURE 3:**
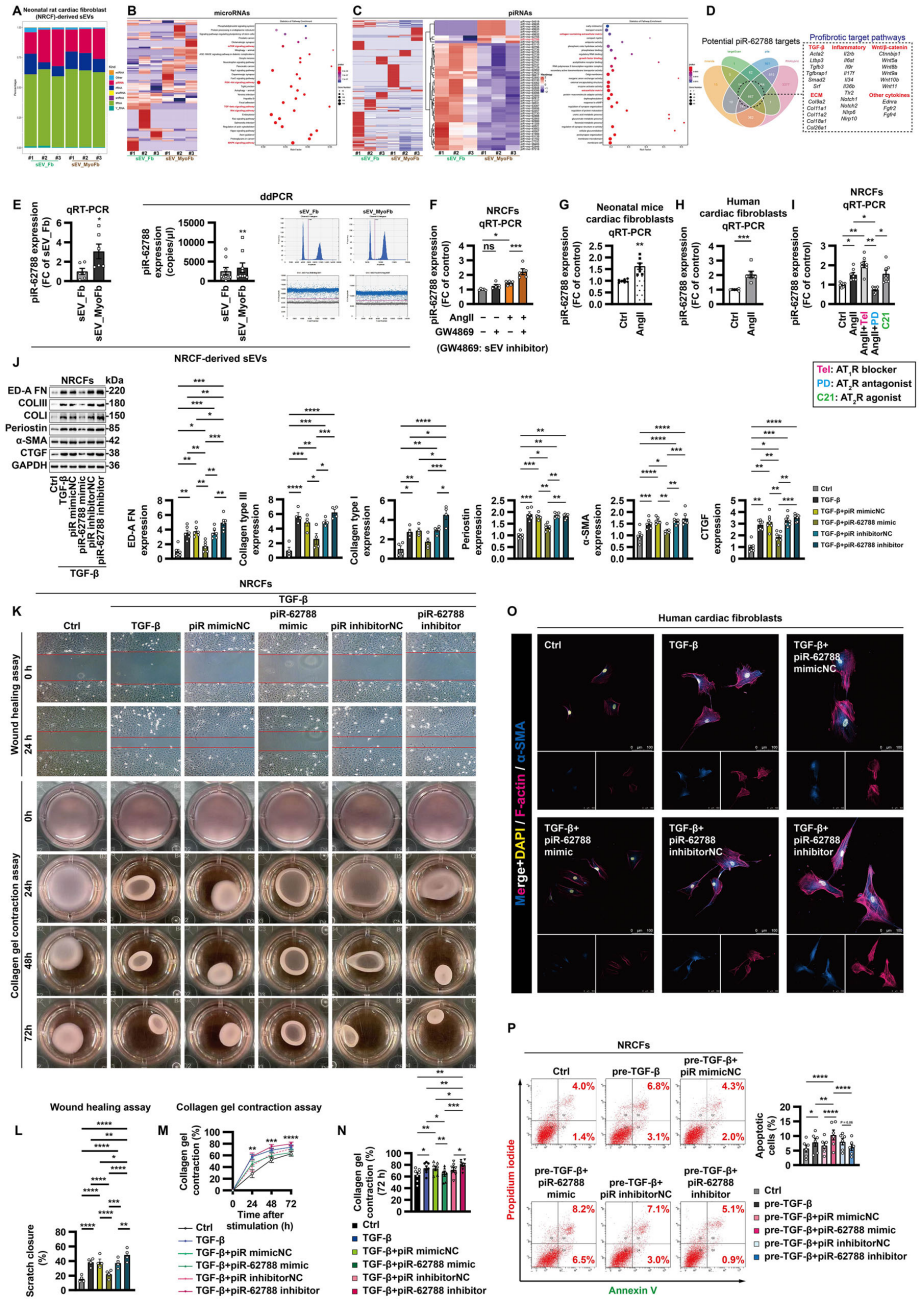
**piR‐62788 is the active element responsible for the anti‐fibrotic effects of MyoFb‐derived sEVs. (A–D)** Small RNA‐sequence analysis of small extracellular vesicles (sEVs). sEVs were isolated from conditioned medium of untreated fibroblasts (Fbs) and angiotensin II (AngII)‐activated myofibroblasts (MyoFbs) by ultracentrifugation. Relative biotype distribution of small RNAs (A). microRNA (miRNA) expression analysis (all miRNAs) and KEGG pathway analysis graph of miRNA target genes (B). Piwi‐interacting RNA (piRNA) expression analysis (all piRNAs), heatmap clustering of the differentially expressed piRNAs significant between sEV_Fb versus sEV_MyoFb, and GO term enrichment graph of upregulated piRNA target genes (C). Potential piR‐62788 target genes based on prediction algorithm (D). **(E)** Expression of piR‐62788 in sEVs was confirmed by qRT‐PCR and droplet digital PCR. **(F)** Effect of GW4869 (10 µM) on piR‐62788 expression in NRCFs. **(G and H)** Validation in neonatal mouse cardiac fibroblasts (G) and human cardiac fibroblasts (H). **(I)** Expression of piR‐62788 in NRCFs after treatment with AngII (100 nM), AngII receptor type 1 (AT_1_R) blocker telmisartan (Tel) (10 µM), AT_2_R antagonist PD123319 (PD) (10 µM) and AT_2_R agonist compound 21 (C21) (10 µM). **(J–N)** NRCFs were transfected with negative control (NC) or piR‐62788 mimic (50 nM) or locked nucleic acid‐based inhibitor (200 nM) for 12 h before exposure to TGF‐β1 (10 ng/mL) for 72 h. Expression of fibrogenic proteins was determined by Western blot (J). Representative photographs of scratch wounds at 0 and 24 h after wounds were made (K), and quantification of migration distances is shown in (L). Collagen pad contraction assay (K, M and N). **(O)** Validation in human cardiac fibroblasts. **(P)** NRCFs were pre‐treated with TGF‐β1 (10 ng/mL) for 72 h. Then, the cells were transfected with NC or piR‐62788 mimic (50 nM) or piR‐62788 inhibitor (200 nM) for 48 h. Annexin V‐positive cells were quantified by flow cytometry. All the qRT‐PCR data are normalized to *U6*. Data shown are mean ± SEM. * *p* < 0.05, ** *p* < 0.01, *** *p* < 0.001, **** *p* < 0.0001. *n* = 3–9, each dot represents a biologically independent sample. Significance was analysed by Student's *t*‐test or one‐way ANOVA followed by Tukey's post hoc test. rRNA, ribosomal RNA; snoRNA, small nucleolar RNA; snRNA, small nuclear RNA; tRNA, transfer RNA; KEGG, Kyoto encyclopaedia of genes and genomes; GO, Gene Ontology; TGF‐β, transforming growth factor‐β; α‐SMA, alpha smooth muscle actin; COL, collagen; CTGF, connective tissue growth factor; ED‐A FN, fibronectin ED‐A domain.

To figure out the underlying mechanisms by which MyoFb‐derived sEVs counterbalance profibrotic signals, we conducted small RNA sequencing from sEVs of untreated fibroblasts and AngII‐activated MyoFbs. sEVs contained various classes of small non‐coding RNAs such as microRNAs (miRNAs). Surprisingly, miRNAs were the least represented biotype, while piRNAs were one of the most abundant biotypes (Figures 3A and S3A). For example, the copy numbers of piR‐62788 were approximately 50 times higher than that of miR‐425‐5p or miR‐450a‐5p (Figure S3B). Therefore, although several fibrosis‐related miRNAs were identified based on previous literature and bioinformatic prediction (Figure 3B), we focused on piRNAs for further investigation.

A total of 1193 distinct piRNAs were found to be present in neonatal rat cardiac fibroblast (NRCF)‐derived sEVs. We identified nine piRNAs were significantly upregulated and 42 piRNAs were downregulated in sEV_MyoFb when compared to sEV_Fb (Figure 3C). Considering that a set of potential target genes for piR‐62788 was involved in the profibrotic signalling pathways (Figure 3D), and that piR‐62788 levels were higher in fibroblasts compared to cardiomyocytes (Figure S3C,D), we selected piR‐62788 as our top candidate.

Expression of sEV‐derived piR‐62788 was confirmed by qRT‐PCR and droplet digital PCR (Figure 3E). Additionally, piR‐62788 levels were increased in fibroblasts in response to AngII, which was further elevated by co‐treatment with exosomal inhibitor GW4869, implying the retention of sEV‐derived piR‐62788 within cells (Figure 3F–H). More importantly, AngII‐induced upregulation of piR‐62788 in fibroblasts was enhanced by AT_1_R blocker telmisartan, while was negated by AT_2_R antagonist PD123319 (Figure 3I).

Next, the regulatory effects of piR‐62788 on fibrotic responses were examined by gain‐ and loss‐of‐function assays. Overexpression of piR‐62788 effectively prevented the expression of fibrogenic proteins, fibroblast migration and collagen gel contraction after TGF‐β1 stimulation; in contrast, knockdown of piR‐62788 accentuated profibrotic activation to some extent (Figure 3J–O). In addition, piR‐62788 induced apoptosis in TGF‐β1‐pre‐treated fibroblasts (Figure 3P). Taken together, the anti‐fibrotic effects of MyoFb‐derived sEVs were at least partly AngII/AT_2_R/piR‐62788‐dependent.

### 3.4 PIWIL2 is Required for the Anti‐Fibrotic Effects of sEV‐Derived piR‐62788

**FIGURE 4:**
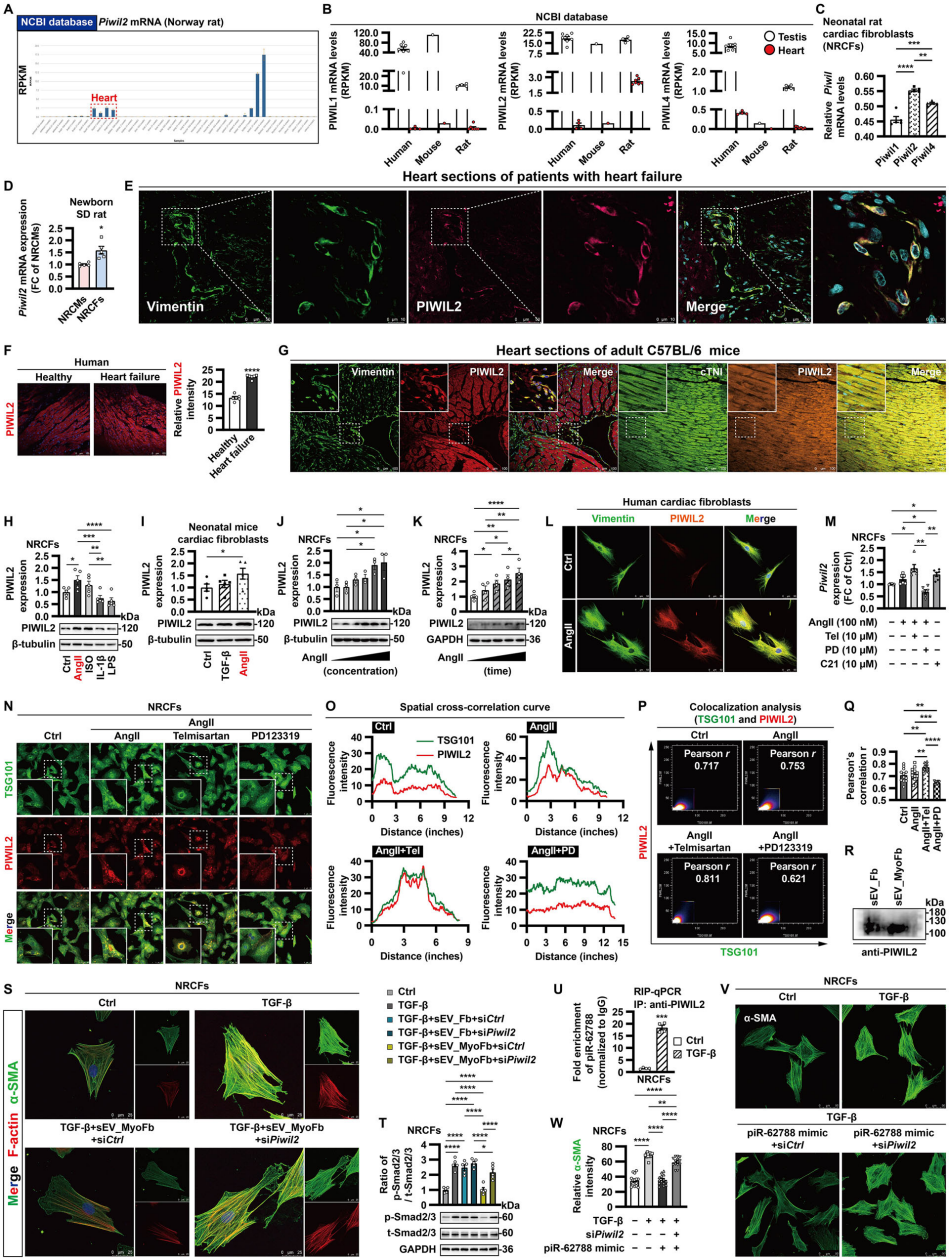
**PIWIL2 is required for the anti‐fibrotic effects of sEV‐derived piR‐62788. (A and B)** Expression of PIWI‐like protein (*Piwil*) mRNA based on RNA‐Seq data downloaded from NCBI database. **(C)** Expression of *Piwil* mRNA in neonatal rat cardiac fibroblasts (NRCFs) was determined by qRT‐PCR. **(D)** Comparison of the expression of *Piwil2* mRNA between neonatal rat cardiomyocytes (NRCMs) and NRCFs. **(E and F)** Immunofluorescence staining showing vimentin (green) and PIWIL2 (red) in human myocardium (E). The average fluorescence intensity of PIWIL2 were quantified (F) (*n* = 1 heart/six different fields in each group). **(G)** Colocalization of PIWIL2 with vimentin or cardiac Troponin I (cTNI) in heart sections of adult mice were visualized by confocal microscopy. **(H and I)** NRCFs or neonatal mice cardiac fibroblasts were treated for 24 h with angiotensin II (AngII) (100 nM), isoproterenol (ISO) (10 µM), transforming growth factor‐β1 (TGF‐β1) (10 ng/mL), interleukin‐1β (IL‐1β) (4 ng/mL), or lipopolysaccharide (LPS) (10 µg/mL). **(J and K)** The dose‐ and time‐dependent expression patterns of PIWIL2 in response to AngII (1 nM, 10 nM, 100 nM, 1 µM and 10 µM; 24, 48, 72 and 96 h) were determined by Western blot. **(L)** Validation in human cardiac fibroblasts. **(M)** Expression of *Piwil2* mRNA in NRCFs after treatment for 24 h with AngII (100 nM), AngII receptor type 1 (AT_1_R) blocker telmisartan (Tel) (10 µM), AT_2_R antagonist PD123319 (PD) (10 µM) and AT_2_R agonist compound 21 (C21) (10 µM). Data are normalized to *Gapdh*. **(N–Q)** NRCFs were stained with TSG101 (green), PIWIL2 (red) and nuclei (DAPI, blue) (N). Colocalization of TSG101 with PIWIL2 was analysed by a spatial cross‐correlation curve (O) and Pearson's correlation test (P and Q) (*n* = 6 biologically independent samples/12 different fields in each group). **(R)** Small extracellular vesicles (sEVs) were isolated from conditioned medium of untreated fibroblasts (Fbs) and AngII‐activated myofibroblasts (MyoFbs) by ultracentrifugation. Expression of PIWIL2 in sEVs was determined by Western blot. **(S and T)** NRCFs transfected with siRNA control (si*Ctrl*) or *Piwil2* siRNA (si*Piwil2*) (50 nM) were co‐cultured with sEV_MyoFb (10 µg/mL) for 6 h before exposure to TGF‐β1 (10 ng/mL). After 72 h, the cells were stained with α‐smooth muscle actin (SMA) (green), F‐actin (red) and nuclei (DAPI, blue) (S); or after 30 min, the cell lysates were harvested for Western blot (T). **(U)** NRCFs were untreated or treated with TGF‐β1 (10 ng/mL) for 24 h. piR‐62788 collected from a RIP‐qPCR assay with cell lysates using an IgG antibody or an anti‐PIWIL2 antibody. **(V and W)** NRCFs were transfected with si*Ctrl* or si*Piwil2* (50 nM) overnight. Then, the cells were treated with piR‐62788 mimic (50 nM) for 12 h before exposure to TGF‐β1 (10 ng/mL). After 72 h, the cells were stained with α‐SMA (green) (V). The average fluorescence intensity of α‐SMA was quantified (W) (*n* = 3 biologically independent samples/14 different fields in each group). Data shown are mean ± SEM. * *p* < 0.05, ** *p* < 0.01, *** *p* < 0.001, **** *p* < 0.0001. *n* = 3–6, each dot represents a biologically independent sample except (F, Q and W). Significance was analysed by Student's *t*‐test or one‐way ANOVA followed by Tukey's post hoc test. TSG101, tumour susceptibility gene 101 protein; RIP, RNA immunoprecipitation.

Then, we tried to identify the piRNA‐associated PIWI protein. Rats have three PIWI homologs including PIWIL1, PIWIL2 and PIWIL4 (Ozata et al. 2019). Intriguingly, even though the preferential expression in testis, *Piwil2* mRNA, but not *Piwil1* or *Piwil4* mRNA, exhibited a relatively higher expression in heart than that in other organ systems (Figure 4A,B), which hinted strongly that PIWIL2 may play a specific role in the cardiovascular system. Indeed, qRT‐PCR validated that *Piwil2* levels were higher in NRCFs when compared with those of *Piwil1* or *Piwil4* (Figure 4C). *Piwil2* levels were lower in neonatal cardiomyocytes than that in fibroblasts (Figure 4D). Immunofluorescence demonstrated the colocalization of vimentin (a marker for mesenchymal cells [mainly fibroblasts]) with PIWIL2 in human heart, and PIWIL2 was upregulated in the myocardium of patients with HF (Figure 4E,F). In normal adult mice hearts, PIWIL2 was expressed in both cardiac fibroblasts and cardiomyocytes (Figure 4G).

We anticipated that PIWIL2 would modulate phenotype and function of cardiac fibroblasts. PIWIL2 was upregulated in response to profibrotic stimuli, while was downregulated in response to proinflammatory cytokines (Figure 4H,I,L). In particularly, in fibroblasts stimulated with AngII, PIWIL2 was induced in a dose‐ and time‐dependent manner (Figure 4J,K). Like piR‐62788, AngII‐induced *Piwil2* expression was forced by AT_1_R blocker telmisartan, whereas inhibited by AT_2_R antagonist PD123319 (Figure 4M). Under basal conditions, *Piwil2* silencing triggered a spontaneously differentiation as demonstrated by prominent stress fibres decorated with α‐SMA (Figure S4A). In pathologic conditions, TGF‐β1‐induced migration, AngII‐promoted proliferation and H_2_O_2_‐stimulated oxidative stress injury were aggravated following *Piwil2* knockdown (Figure S4B–G).

Additionally, immunofluorescence showed PIWIL2 was primarily present in the cytoplasm of fibroblasts; colocalization of PIWIL2 with sEV‐associated marker TSG101 was boosted by telmisartan, whereas restrained by PD123319 (Figure 4N–Q), indicating that AngII/AT_2_R signalling provoked the release of PIWIL2‐containing sEVs. Meanwhile, Western blot also confirmed the presence of PIWIL2 protein in NRCF‐derived sEVs (Figure 4R).

Furthermore, we explored whether PIWIL2 was required for the anti‐fibrotic actions of piR‐62788. MyoFb‐derived sEVs attenuated incorporation of α‐SMA into cytoskeletal stress fibres and activation of Smad2/3 signalling, which were abrogated after *Piwil2* knockdown (Figure 4S,T). A specific interaction between PIWIL2 and piR‐62788 was verified by RNA immunoprecipitation (RIP)‐qPCR assay, and this association was markedly reinforced upon TGF‐β1 stimulation (Figure 4U). Notably, the suppressive effect of piR‐62788 mimic on TGF‐β1‐induced α‐SMA expression was largely blocked after treatment with *Piwil2* siRNA (Figure 4V,W). To sum up, sEV‐derived piR‐62788 was loaded into PIWIL2 and formed a complex to participate in the regulation of fibrogenesis.

### 3.5 piR‐62788/PIWIL2 Complex Blocks TGF‐β1‐Induced SRF Upregulation Through Interacting With *Srf* mRNA

**FIGURE 5:**
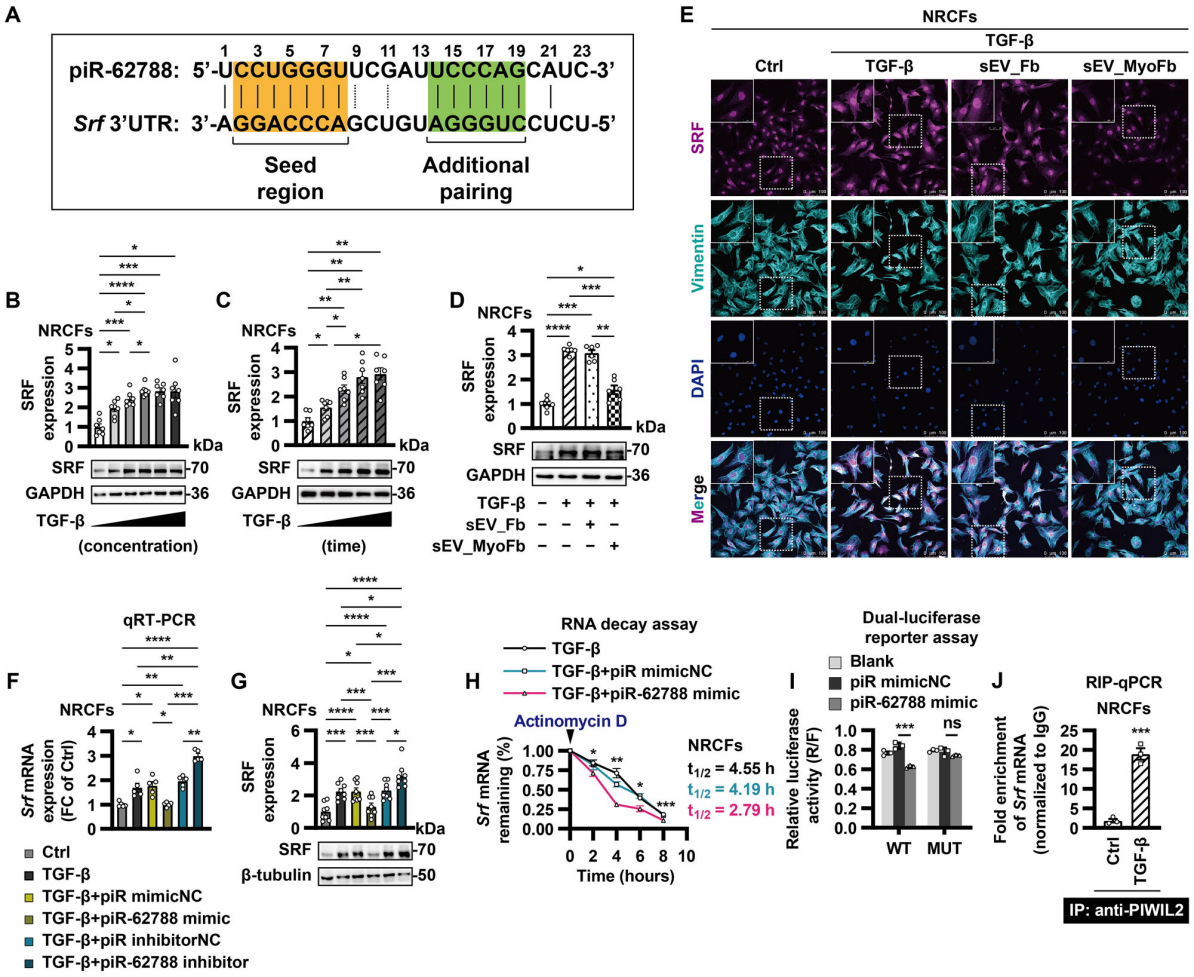
**piR‐62788/PIWIL2 complex blocks TGF‐β1‐induced SRF upregulation through interacting with *Srf* mRNA. (A)** A schematic representation of the binding site between Piwi‐interacting RNA (piR)‐62788 and 3’ untranslated region (UTR) of serum response factor (*Srf*) mRNA. **(B and C)** The dose‐ and time‐dependent expression patterns of SRF in neonatal rat cardiac fibroblasts (NRCFs) in response to TGF‐β1 (0.5, 1, 5, 10 and 20 ng/mL; 24, 48, 72 and 96 h) were determined by Western blot. **(D and E)** Small extracellular vesicles (sEVs) were isolated from conditioned medium of untreated fibroblasts (Fbs) and angiotensin II‐activated myofibroblasts (MyoFbs) by ultracentrifugation. NRCFs were co‐cultured with sEVs (10 µg/mL) for 6 h before exposure to TGF‐β1 (10 ng/mL) for 72 h. Then, the cells were harvested for Western blot (D); or stained with SRF (purple), vimentin (cyan) and nuclei (DAPI, blue) (E). **(F and G)** NRCFs were transfected with negative control (NC) or piR‐62788 mimic (50 nM) or locked nucleic acid‐based inhibitor (200 nM) for 12 h before exposure to TGF‐β1 (10 ng/mL). Expression of SRF was determined by qRT‐PCR (F) and Western blot (G). **(H)** NRCFs were transfected with NC or piR‐62788 mimic (50 nM) for 12 h. Then, the cells were treated with TGF‐β1 (10 ng/mL) plus actinomycin D (5 µg/mL). Expression of *Srf* mRNA was determined by qRT‐PCR, and its half‐life (t_1/2_) was measured (*n* = 5 biologically independent samples per group). **(I)** The luciferase vector cloned with *Srf* mRNA 3’UTR wild‐type (WT) or mutant (MUT) sequence or empty vector (blank) was co‐transfected with NC or piR‐62788 mimic (50 nM). **(J)** NRCFs were untreated or treated with TGF‐β1 (10 ng/mL) for 24 h. *Srf* mRNA collected from a RIP‐qPCR assay with cell lysates using an IgG antibody or an anti‐PIWIL2 antibody. Data shown are mean ± SEM. * *p* < 0.05, ** *p* < 0.01, *** *p* < 0.001, **** *p* < 0.0001. *n* = 3–8, each dot represents a biologically independent sample. Significance was analysed by Student's *t*‐test or one‐way ANOVA followed by Tukey's post hoc test. TGF‐β, transforming growth factor‐β; RIP, RNA immunoprecipitation; α‐SMA, alpha smooth muscle actin; CTGF, connective tissue growth factor; PIWIL2, PIWI‐like protein 2.

To dissect the molecular mechanisms by which piR‐62788/PIWIL2 complex modulates fibrogenesis, bioinformatics analysis was conducted using TargetScan, miRanda, PITA and RNAhybrid. Certainly, several profibrotic genes were predicted to be potential target genes of piR‐62788 (Figure 3D), but we found 3′ untranslated region (UTR) of serum response factor (*Srf*) mRNA was perfectly complementary to piR‐62788 (Figure 5A). Since the activation of transcription factor SRF occurs downstream of TGF‐β1, and plays a major role in MyoFb differentiation (Zhang et al. 2001; Small et al. 2010), we selected SRF for further study.

Treatment with TGF‐β1 resulted in a dose‐ and time‐dependent SRF upregulation in fibroblasts (Figure 5B,C). TGF‐β1‐induced increase in SRF was restrained by MyoFb‐derived sEVs (Figure 5D,E). Consistently, TGF‐β1‐stimulated SRF expression was turned down by piR‐62788 overexpression, while enhanced by piR‐62788 knockdown at both protein and mRNA levels (Figure 5F,G). RNA decay assay showed that piR‐62788 overexpression decreased the stability of *Srf* mRNA in TGF‐β1‐treated fibroblasts (Figure 5H). As expected, piR‐62788 mimic reduced the luciferase activities of the wild‐type (WT) reporter vector but not mutant (MUT) reporter vector (Figure 5I), supporting a direct binding between piR‐62788 and 3′UTR of *Srf* mRNA. Besides, *Srf* mRNA was detectable in PIWIL2 immunoprecipitates, especially upon stimulation with TGF‐β1 (Figure 5J). These findings suggested that SRF was a direct downstream target of piR‐62788.

### 3.6 sEV‐Encapsulated piR‐62788 Protects From Pressure‐Overload‐Induced Cardiac Remodelling

**FIGURE 6:**
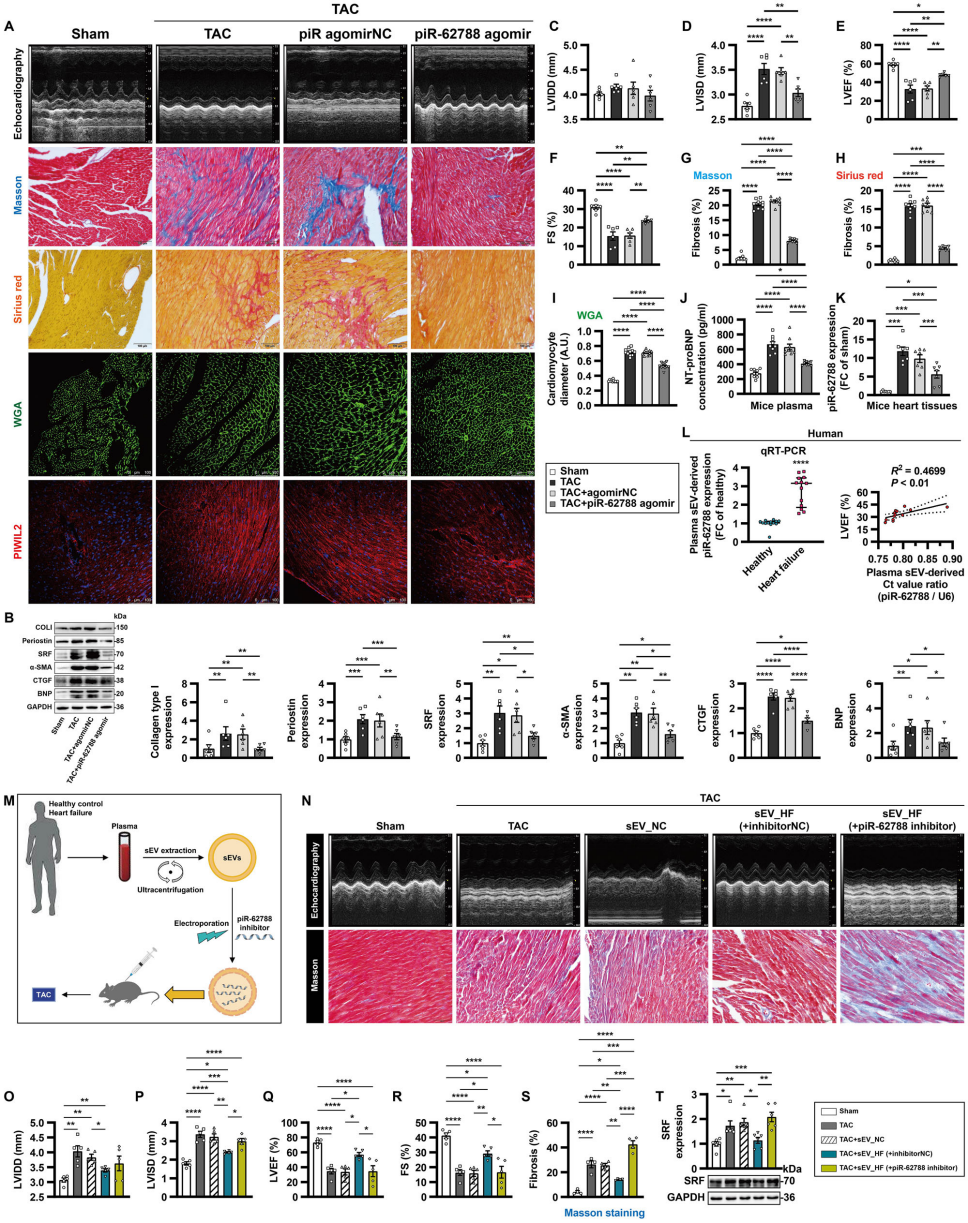
**sEV‐encapsulated piR‐62788 protects from pressure‐overload‐induced cardiac remodelling. (A–K)** For Piwi‐interacting RNA (piR)‐62788 overexpression, mice were treated immediately after surgery with a tail vein injection of negative control (NC) or piR‐62788 agomir (2.5 nmol) every week. Various analyses were performed 4 weeks after the transverse aortic constriction (TAC) surgery or sham operation. Representative images of echocardiography and quantitative analysis (A, and C–F). Expression of cardiac remodelling‐related proteins was determined by Western blot (B). Masson's staining and picrosirius red staining were used to determine myocardial fibrosis (A, G and H) (*n* = 4 hearts/8–9 different fields in each group). Representative images of wheat germ agglutinin (WGA)‐stained sections and measurement of cardiomyocyte diameter (A and I) (*n* = 5 hearts/11 different fields in each group). Immunofluorescence staining showing PIWIL2 (red) (A). Plasma N‐terminal pro‐BNP (NT‐proBNP) concentration was measured by ELISA (J). Expression of piR‐62788 in mice heart tissues was determined by qRT‐PCR (K). **(L)** Small extracellular vesicles (sEVs) were isolated from plasma of healthy subjects and patients with heart failure using ultracentrifugation. Expression of piR‐62788 was determined by qRT‐PCR (left panel). Correlation between LVEF and piR‐62788 in patients with heart failure was analysed by Pearson correlation test (right panel). **(M–T)** in vivo sEV treatment. Experimental setup (M). sEVs were isolated from plasma of healthy subjects (sEV_NC) and patients with heart failure (sEV_HF) using ultracentrifugation. Locked nucleic acid (LNA)‐based piR‐62788 inhibitor or scrambled negative control (NC) was transfected into sEV_HF utilizing electroporation. Mice were intramyocardially injected sEVs during surgery. Representative images of echocardiography and quantitative analysis (N–R). Masson's staining was used to determine myocardial fibrosis (N and S). Expression of serum response factor (SRF) was determined by Western blot (T). All the qRT‐PCR data are normalized to *U6*. Data shown are mean ± SEM. * *p* < 0.05, ** *p* < 0.01, *** *p* < 0.001, **** *p* < 0.0001. *n* = 4–13, each dot represents a biologically independent sample except (G–I). Significance was analysed by Student's *t*‐test or one‐way ANOVA followed by Tukey's post hoc test. LVIDD, left ventricular internal diameter at end‐diastole; LVISD, left ventricular internal diameter at end‐systole; LVEF, left ventricular ejection fraction; FS, fractional shortening; PIWIL, PIWI‐like protein; α‐SMA, alpha smooth muscle actin; COL, collagen; CTGF, connective tissue growth factor. BNP, brain natriuretic peptide.

To gain insight into the role of piR‐62788 in modulating cardiac remodelling in vivo, TAC‐induced chronic HF was established. Myocardial piR‐62788 levels were upregulated after 28 days of TAC (Figure 6K). Overexpression of piR‐62788 by agomir resulted in reduced interstitial fibrosis, downregulated fibrogenic proteins, attenuated cardiac hypertrophy and improved systolic function in pressure‐overload heart (Figure 6A–J).

In addition, plasma sEV‐encapsulated piR‐62788 was dramatically upregulated in HF patients and negatively correlated with left ventricular ejection fraction (Figure 6L). In order to further examine the clinical translational relevance of piR‐62788, we injected plasma‐derived sEVs into TAC mice (Figure 6M). sEVs isolated from the plasma of HF patients protected the pressure‐overload heart from adverse remodelling. In contrast, manipulation of sEV‐piR‐62788 using locked nucleic acid (LNA)‐based inhibitor impaired its anti‐fibrotic activities (Figure 6N–T).

## 4 Discussion

In response to acute injury or chronic stress, phenotypic transition of fibroblasts is triggered by various profibrotic cytokines and proinflammatory factors such as AngII, TGF‐β1 and IL‐1β (Younesi et al. 2024; Henderson et al. 2020). Meanwhile, the fibrogenic responses have shown to be self‐limited owing to activation of negative inhibitory pathways. AngII is capable of inducing endogenous repressors of fibrosis such as adrenomedullin (Tsuruda et al. 1999), calcitonin gene‐related peptide (Li et al. 2020), activating transcription factor 3 (Li et al. 2017) and Smad7 (Humeres et al. 2024). Given that fibroblasts display substantial dynamic plasticity and functional heterogeneity (Younesi et al. 2024; Henderson et al. 2020), we speculated that these anti‐fibrotic signals were associated with different functional states of the same fibroblast subtype and (or) phenotypically distinct fibroblast populations. For instance, atrial fibroblasts are more sensitive to profibrotic cytokines than ventricular fibroblasts (Burstein et al. 2008). McLellan et al. identify *Cilp* (cartilage intermediate layer protein)^+^ cardiac fibroblasts emerge as the predominant fibroblast subpopulation following AngII infusion (McLellan et al. 2020). Surprisingly, CILP1 is upregulated in multiple cardiovascular conditions and can ameliorate cardiac remodelling through interfering TGF‐β1 signalling (Zhang et al. 2018; Barallobre‐Barreiro et al. 2012; van Nieuwenhoven et al. 2017). Unfortunately, we did not identify the specific fibroblast subset(s) in the present study. This is an interesting issue and worthy of further investigation.

Previous studies demonstrate that activated lung fibroblasts produce sEVs with anti‐fibrotic molecules encapsulated inside (Lacy et al. 2019; Martin‐Medina et al. 2018). Co‐culture with cardiac fibroblast‐derived sEVs leads to AT_2_R upregulation in cardiomyocytes (Lyu et al. 2015). Exosomal inhibitor GW4869 has been shown to reduce inflammation, atherosclerosis and pathological fibrosis in vivo (Lyu et al. 2015; Lallemand et al. 2018). Our work also implicated the anti‐fibrotic actions of GW4869 in vitro, which can be explained by sequestration of instrumental molecules within fibroblasts. Considering that of three canonical profibrotic stimuli (AngII, TGF‐β1 and endothelin‐1), only Ang II promotes sEV release from cardiac fibroblasts (Lyu et al. 2015), we tested the sEVs secreted by AngII‐treated MyoFbs. We are the first to show that sEVs of AngII‐activated MyoFbs were able to counterbalance profibrotic activation in vitro. This finding was also validated in vivo. Introduction of sEVs isolated from plasma of HF patients into mice restricted fibrotic remodelling and dysfunction following TAC. It has been suggested that sEVs of cardiac stromal cells isolated from HF patients contain decreased profibrotic miR‐21 as compared with healthy sEVs (Qiao et al. 2019; Thum et al. 2008). A recent study revealed that EVs secreted by human heart‐derived cells possess anti‐inflammatory and anti‐fibrotic properties (Parent et al. 2024). Therefore, sEVs collected from MyoFbs or chronic HF patients may hold captivating therapeutic potential for cardiac fibrosis.

It has been well documented that miRNA plays a pivotal role in modulating cardiac remodelling. Indeed, several fibrosis‐linked miRNAs were upregulated in MyoFb‐derived sEVs in our experiments. Curiously, piRNAs were found to be substantially enriched in sEVs. piRNA‐mediated post‐transcriptional gene silencing was initially discovered to be associated with a negative autogenous control of gene expression (Aravin et al. 2001). This notion is further supported by the evidence that a large proportion of piRNAs correspond to the 3′UTRs of an extensive set of mRNAs (Yamtich et al. 2015; Robine et al. 2009). Our results demonstrated that most piRNAs are downregulated in MyoFb‐derived sEVs, in agreement with the observation that 585 piRNAs were upregulated and 4,623 piRNAs were downregulated in serum‐derived sEVs of HF patients (Yang et al. 2018). We focused on the upregulated piRNAs and identified piR‐62788 as a potent endogenous suppressor of fibrogenic activation. Notably, sEVs encapsulated with piR‐62788 inhibitor further accentuated myocardial fibrosis as compared with TAC group, which could be explained by the loss‐of‐function of endogenous piR‐62788.

Generally, PIWI proteins are required for piRNA‐induced cleavage of target mRNA. PIWIL4 has been identified as a functional partner of piRNAs in cardiomyocytes (Gao et al. 2020). Mouse MIWI2 (PIWIL4) prohibits fibroblast transdifferentiation (Shi et al. 2019). PIWIL2 represses circadian rhythms in testis and cancer cells (Lu et al. 2017). Nevertheless, to our knowledge, the potential impacts of PIWI on fibroblast biology have not been clarified comprehensively. Our data showed that PIWIL2 was highly responsive to various stresses in cultured fibroblasts, indicative of its disease‐specific regulatory roles. Furthermore, we demonstrated that PIWIL2 formed a functional complex with piR‐62788 and was essential for the anti‐fibrotic actions of piR‐62788. Of note, pharmacological interventions of AngII receptor remarkably influenced both piR‐62788 and PIWIL2, providing an innovative understanding of the protective role of AngII/AT_2_R signalling.

MRTF/SRF signalling axis is crucial for pathological cardiac remodelling by inducing numerous hypertrophic and fibrogenic genes such as *Myh7*, *Ctgf*, *Col1a1*, *Col3a1* and *Acta2* (Zhang et al. 2001; Small et al. 2010; Sandbo et al. 2009). SRF, a member of the MADS (MCM1, Agamous, Deficiens and SRF) box‐containing family of transcription factors, plays a primary role in the regulation of fibroblast activation, migration and apoptosis via binding to a conserved CArG sequence (CC[A/T]_6_GG) termed serum response element (Lighthouse and Small 2016; Davis and Molkentin 2014). SRF inhibitor‐containing nanoparticles effectively alleviate pulmonary fibrosis (Knipe et al. 2023). The activity and expression of SRF have also shown to be profoundly affected by several classes of endogenous noncoding RNAs such as miR‐133 (Chen et al. 2006), miR‐664‐5p (Cai et al. 2018), circular RNA *circSlc8a1* (Lim et al. 2019), long noncoding RNA (lncRNA) CARDINAL (Anderson et al. 2021) and lncRNA CARMN (Ni et al. 2021; Liu et al. 2024). Herein, we characterized a novel sEV‐derived piRNA/PIWI machinery in regulation of SRF signalling. Broadly speaking, piRNAs guide endonuclease PIWI sub‐clade to destabilize and cleave their mRNA targets through a miRNA‐like mechanism (Ozata et al. 2019; Shi et al. 2022). sEV‐mediated transport of functional piR‐62788/PIWIL2 complexes would allow a fast response to TGF‐β1/SRF‐dependent fibrogenic signal.

In summary, the present study uncovered a novel negative feedback loop controlled by cardiac MyoFb‐derived sEVs. Induction of sEV‐encapsulated piR‐62788 in AngII/AT_2_R‐stimulated fibroblasts restrains a matrix‐synthesizing and invasive migratory phenotype through piR‐62788/PIWIL2 complex‐mediated *Srf* degradation. This work opens the way for developing strategies that protect from adverse cardiac fibrotic remodeling.

## Author Contributions

**Shichao Li**: conceptualization, data curation, formal analysis, investigation, methodology, validation, visualization, writing ‐ original draft, writing ‐ review and editing. **Shuwen Su**: formal analysis, investigation, validation, project administration. **Gaopeng Xian**: formal analysis, investigation, methodology, validation, project administration. **Shunyi Li**: formal analysis, investigation, validation, software. **Guoheng Zhong**: methodology, resources, software, visualization. **Liming Wen**: methodology, software, visualization. **Dingli Xu**: conceptualization, data curation, project administration, supervision, funding acquisition, resources, writing ‐ review and editing. **Qingchun Zeng**: conceptualization, data curation, project administration, supervision, funding acquisition, resources, writing ‐ review and editing.

## Conflicts of Interest

The authors declare no competing interests.

## Supporting information




**Supplementary Material**: jev270204‐sup‐0001‐SuppMat.docx

## Data Availability

All data generated for this study are available from the corresponding authors upon reasonable request.
