# Supplementary material for: Cardiac Myofibroblast‐Derived Small Extracellular Vesicles Moderate Fibrotic Responses via piRNA‐62788/PIWIL2‐Mediated SRF Silencing
Source: J Extracell Vesicles. 2025 Nov 28;14(12):e70204. doi: 10.1002/jev2.70204 (PMC12661122; doi:10.1002/jev2.70204)
Supplement: Supplementary file 1 — Supplementary Material: jev270204‐sup‐0001‐SuppMat.docx [file JEV2-14-e70204-s001.docx]

**Supplementary Materials for**

**Cardiac myofibroblast-derived small extracellular vesicles moderate fibrotic responses via piRNA-62788/PIWIL2-mediated SRF silencing**

Shichao Li, Shuwen Su, Gaopeng Xian, Shunyi Li, Guoheng Zhong, Liming Wen, Dingli Xu^*^, Qingchun Zeng^*^

^*^Corresponding author. E-mail addresses: dinglixu@smu.edu.cn (D. Xu); qingchunzeng@smu.edu.cn (Q. Zeng)

State Key Laboratory of Organ Failure Research, Department of Cardiology, Nanfang Hospital, Southern Medical University, Guangzhou 510515, China.

Guangdong Provincial Key Laboratory of Cardiac Function and Microcirculation, Southern Medical University, Guangzhou 510515, China.

**This PDF file includes:**

Figures. S1 to S5

Tables. S1 to S4





**Figure S1. Quantitative analysis of sEV uptake.** Primary cardiac fibroblasts (NRCFs) were isolated and cultured from neonatal rats. Small extracellular vesicles (sEVs) were isolated from conditioned medium of untreated fibroblasts (Fbs) and angiotensin II-activated myofibroblasts (MyoFbs) by ultracentrifugation. **(A)** Both sEV_Fb and sEV_MyoFb were labeled with PKH67 dye (green). NRCFs were co-cultured with either sEV_Fb or sEV_MyoFb (10 μg/ml) for 16 hours. Immunofluorescence staining showing vimentin (red). **(B–D)** Both sEV_Fb and sEV_MyoFb were labeled with PKH67 dye (green). NRCFs were untreated or pre-treated with transforming growth factor-β1 (TGF-β1) (10 ng/ml) for 72 hours. Then, the cells were co-cultured with either sEV_Fb or sEV_MyoFb (10 μg/ml) for 16 hours. The uptake efficiency was determined using flow cytometry (B), and was quantified as the percentage of PKH-positive cells (C) or mean fluorescence intensity (D). **(E–G)** sEV_Fb and sEV_MyoFb were labeled with PKH67 (green) and PKH26 (red), respectively. NRCFs were untreated or pre-treated with TGF-β1 (10 ng/ml) for 72 hours. Equal amounts of PKH67-labeled sEV_Fb and PKH26-labeled sEV_MyoFb were mixed before co-culture with NRCFs for 16 hours. The uptake efficiency was determined using flow cytometry (E), and was quantified as the frequency ([PKH-positive cells/total cells] × 100%) (F) or mean fluorescence intensity (G). **(H–J)** Comparative analysis of sEV uptake in different cell types. Experimental setup (H). NRCF-derived sEVs were isolated from conditioned medium by ultracentrifugation and labeled with PKH67 dye (green). Primary heart cells were isolated from neonatal rats and grown on glass coverslips for 24 hours before incubation with PKH67-labeled sEVs for another 6 hours. Then, the cells were subjected to immunofluorescence staining. Internalization of PKH67-labeled sEVs by cells was quantified based on their relative fluorescence intensity using confocal laser scanning microscope (I and J) (*n* = 3 biologically independent samples/10 different fields in each group). Data shown are mean ± SEM. * *p* < 0.05, ** *p* < 0.01. *n* = 3–8, each dot represents a biologically independent sample except (J). Significance was analyzed by Student’s *t*-test or one-way ANOVA followed by Tukey’s *post hoc* test. cTNI, cardiac Troponin I (cardiomyocyte marker); α-SMA, alpha-smooth muscle actin (smooth muscle marker).


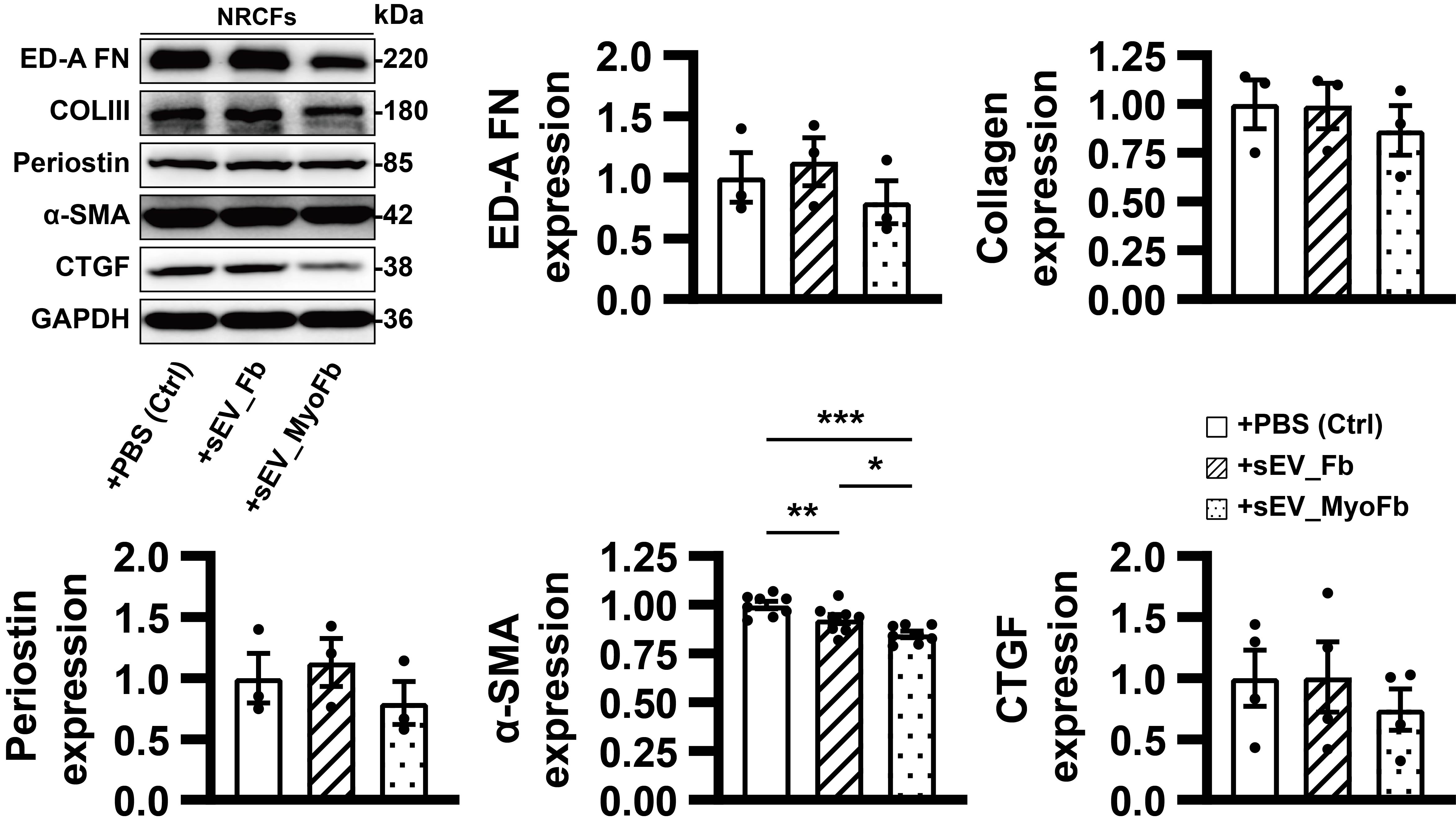


**Figure S2. Effects of cardiac fibroblast-derived sEVs on fibroblast phenotype under basal conditions.** Primary cardiac fibroblasts (NRCFs) were isolated and cultured from neonatal rats. Small extracellular vesicles (sEVs) were isolated from conditioned medium of untreated fibroblasts (Fbs) and angiotensin II-activated myofibroblasts (MyoFbs) by ultracentrifugation. NRCFs were co-cultured with sEVs (10 μg/ml) for 48 hours. Expression of fibrogenic proteins was determined by Western blot. Data shown are mean ± SEM. * *p* < 0.05, ** *p* < 0.01, *** *p* < 0.001. *n* = 3–8, each dot represents a biologically independent sample. Significance was analyzed by one-way ANOVA followed by Tukey’s *post hoc* test. α-SMA, alpha-smooth muscle actin; COL, collagen; CTGF, connective tissue growth factor; ED-A FN, fibronectin ED-A domain.


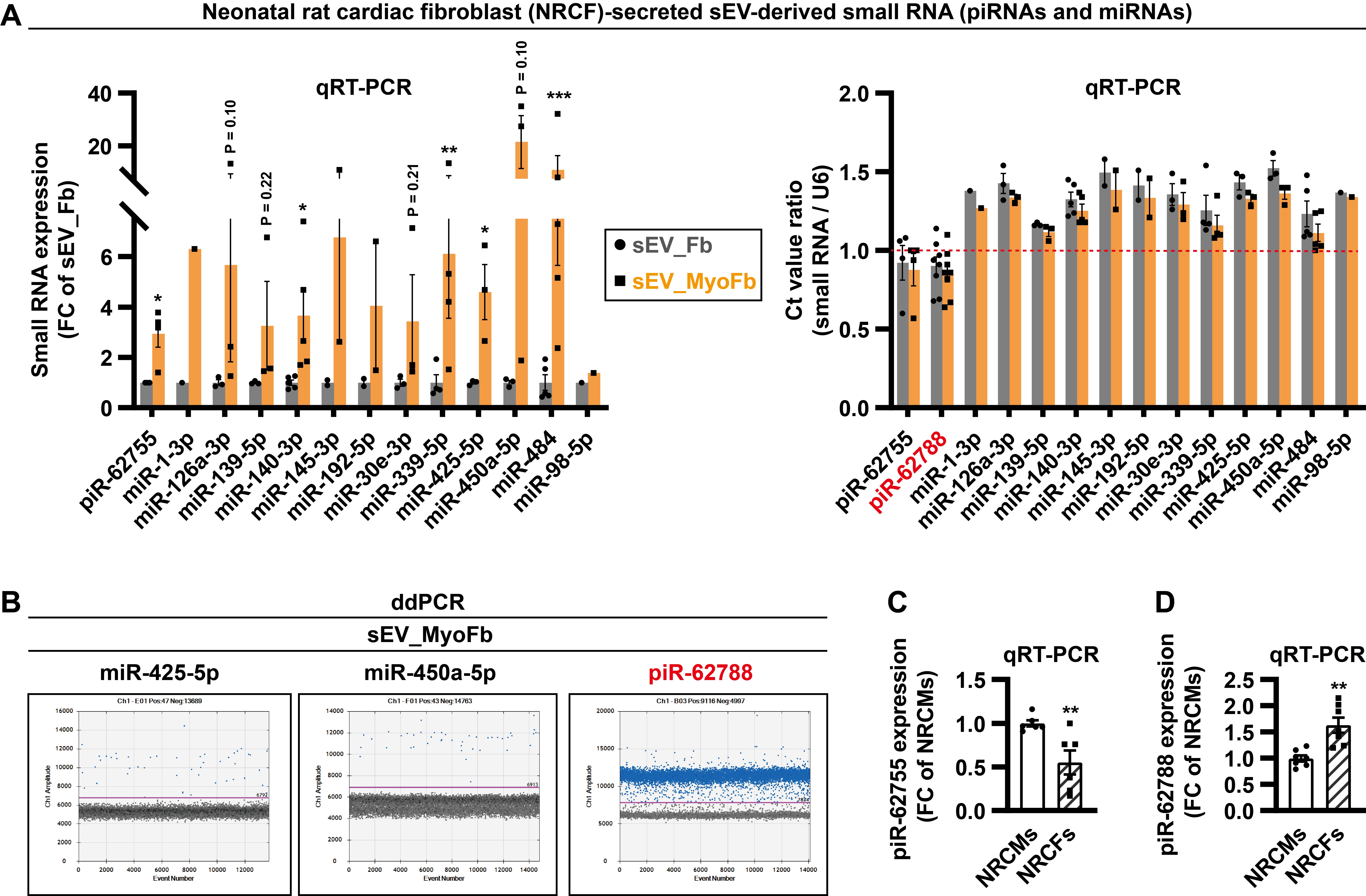


**Figure S3. Verification of small RNA-seq.** Primary cardiac fibroblasts (NRCFs) were isolated and cultured from neonatal rats. Small extracellular vesicles (sEVs) were isolated from conditioned medium of untreated fibroblasts (Fbs) and angiotensin II-activated myofibroblasts (MyoFbs) by ultracentrifugation. **(A)** Validation of differentially expressed microRNAs (miRNAs) and Piwi-interacting RNAs (piRNAs) using qRT-PCR. **(B)** Expression of miR-425-5p, miR-450a-5p, and piR-62788 in sEV_MyoFb was determined by droplet digital PCR. **(C and D)** Comparison of the expression of piR-62755 and piR-62788 between NRCFs and cardiomyocytes (neonatal rat cardiomyocytes [NRCMs]). All the qRT-PCR data are normalized to *U6*. Data shown are mean ± SEM. * *p* < 0.05, ** *p* < 0.01, *** *p* < 0.001. *n* = 1–8, each dot represents a biologically independent sample. Significance was analyzed by Student’s *t*-test.





**Figure S4. PIWIL2 modulates fibroblast phenotype and function *in vitro*. (A)** Neonatal rat cardiac fibroblasts (NRCFs) were transfected with siRNA control (si*Ctrl*) or *Piwil2* siRNA (si*Piwil2*) (50 nM) for 48 hours. Then, the cells were stained with alpha smooth muscle actin (α-SMA) (green), F-actin (red), and nuclei (DAPI, blue). **(B and C)** NRCFs were transfected with si*Ctrl* or si*Piwil2* (50 nM). Then, the cells were treated with TGF-β1 (10 ng/ml), representative photographs of scratch wounds at 0 and 24 hours after wounds were made (B), and quantification of migration distances is shown in (C). **(D and E)** NRCFs were transfected with si*Piwil2* before exposure to angiotensin II (AngII) (100 nM) for 24 hours. Proliferative capacity was assessed by EdU incorporation assay (*n* = 4 biologically independent samples/8 different fields in each group). **(F and G)** NRCFs were transfected with si*Piwil2* before exposure to H_2_O_2_ (100 μM) for 24 hours. After loading with JC-10 dye (a mitochondrial membrane potential probe), images were acquired using microscope and the mean fluorescence intensity ratio of monomers (green) to aggregates (red) was calculated (H). Data shown are mean ± SEM. * *p* < 0.05, ** *p* < 0.01, *** *p* < 0.001, **** *p* < 0.0001. *n* = 4–6, each dot represents a biologically independent sample except (E). Significance was analyzed by one-way ANOVA followed by Tukey’s *post hoc* test. PIWIL, PIWI-like protein; TGF-β, transforming growth factor-β; EdU, 5-ethynyl-2’-deoxyuridine.


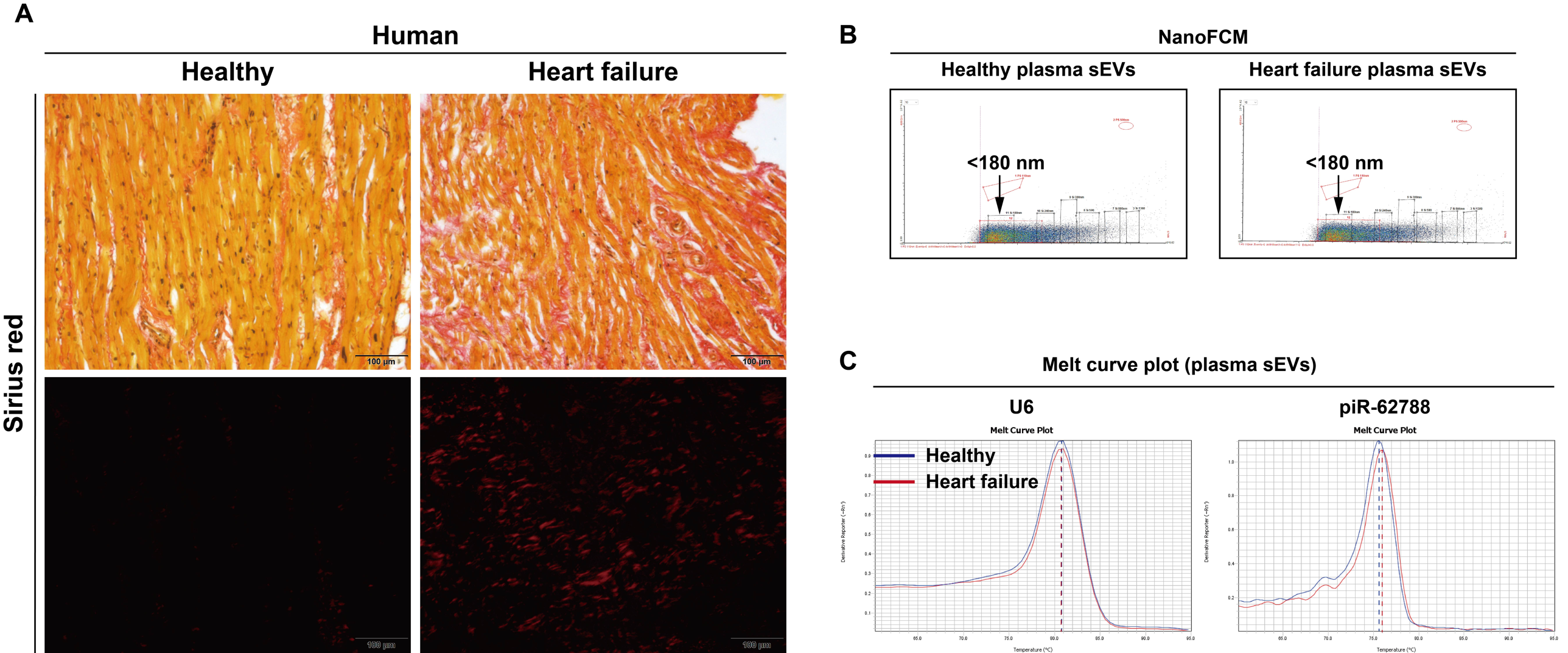


**Figure S5. Analysis of sEVs isolated from human plasma.** Small extracellular vesicles (sEVs) were isolated from plasma of healthy controls and patients with heart failure using ultracentrifugation. **(A)** validation of the collagen deposition in failing hearts. **(B)** sEV characterization was confirmed by nano-flow cytometry (NanoFCM) (*n* = 3 biologically independent samples per group). **(C)** Melting curves were established for qRT-PCR. piRNA, Piwi-interacting RNA.

**Table S1. Primary antibodies used in this study**

| **Target** | **Vendor** | **Catalog** | **Dilution** |
| --- | --- | --- | --- |
| α-SMA | Abcam | ab124964 | 1:10000 (WB) |
|  |  |  | 1:500 (ICC) |
|  |  |  | 1:2000 (IHC) |
| β-actin | Abcam | ab8224 | 1:1000 (WB) |
| BNP | ABclonal | A2179 | 1:1000 (WB) |
| β-tubulin | Abcam | Ab179513 | 1:5000 (WB) |
| Calnexin | Santa Cruz | sc-23954 | 1:200 (WB) |
| CD63 | Santa Cruz | sc-5275 | 1:200 (WB) |
| Collagen type I | Proteintech | 67288-1-Ig | 1:5000 (WB) |
| Collagen type III | Proteintech | 22734-1-AP | 1:1000 (WB) |
| CTGF | Santa Cruz | sc-101586 | 1:200 (WB) |
|  |  |  | 1:100 (ICC) |
| cTNI | Proteintech | 21652-1-AP | 1:200 (ICC & IHC) |
| ED-A FN | Santa Cruz | sc-59826 | 1:500 (WB) |
| GAPDH | Abcam | ab181602 | 1:10000 (WB) |
| Periostin | Proteintech | 66491-1-Ig | 1:2500 (WB) |
| Phalloidin | Abcam | ab176757 | 1:1000 (ICC) |
| PIWIL2 | Santa Cruz | sc-377258 | 1:200 (WB) |
|  |  |  | 1:100 (ICC & IHC) |
|  |  |  | 5 μg (RIP) |
| p-Smad2/3 | Cell Signaling Technology | 8828 | 1:1000 (WB) |
| Smad2/3 | Cell Signaling Technology | 8685 | 1:1000 (WB) |
| SRF | Proteintech | 16821-1-AP | 1:5000 (WB) |
|  |  |  | 1:500 (ICC) |
| TSG101 | Santa Cruz | sc-7964 | 1:200 (WB) |
|  | Proteintech | 28283-1-AP | 1:200 (ICC) |
| Vimentin | HUABIO | ET1610-39 | 1:200 (ICC & IHC) |

**Table S2. The primer sequences used for qRT-PCR in this study**

| **Genes** | **Sequences** |
| --- | --- |
| miR-1-3p | F: gcgggTGGAATGTAAAGAAGTGTGTAT |
|  | R: universal primer (Accurate Biology, Cat#: AG11716) |
| miR-126a-3p | F: ccgTCGTACCGTGAGTAATAATGCG |
|  | R: universal primer (Accurate Biology, Cat#: AG11716) |
| miR-139-5p | F: TCTACAGTGCACGTGTCTCCAG |
|  | R: universal primer (Accurate Biology, Cat#: AG11716) |
| miR-140-3p | F: TACCACAGGGTAGAACCACGG |
|  | R: universal primer (Accurate Biology, Cat#: AG11716) |
| miR-145-3p | F: ccggGGATTCCTGGAAATACTGTTC |
|  | R: universal primer (Accurate Biology, Cat#: AG11716) |
| miR-192-5p | F: cgcCTGACCTATGAATTGACAGCC |
|  | R: universal primer (Accurate Biology, Cat#: AG11716) |
| miR-30e-3p | F: aggCTTTCAGTCGGATGTTTACAGC |
|  | R: universal primer (Accurate Biology, Cat#: AG11716) |
| miR-339-5p | F: TCCCTGTCCTCCAGGAGC |
|  | R: universal primer (Accurate Biology, Cat#: AG11716) |
| miR-425-5p | F: AATGACACGATCACTCCCGTTGA |
|  | R: universal primer (Accurate Biology, Cat#: AG11716) |
| miR-450a-5p | F: cgggTTTTGCGATGTGTTCCTAATGT |
|  | R: universal primer (Accurate Biology, Cat#: AG11716) |
| miR-484 | F: TCAGGCTCAGTCCCCTCC |
|  | R: universal primer (Accurate Biology, Cat#: AG11716) |
| miR-98-5p | F: gcgggTGAGGTAGTAAGTTGTATTGTT |
|  | R: universal primer (Accurate Biology, Cat#: AG11716) |
| piR-62755 | F: cgggGGTATAGTGGTGAGCATAG |
|  | R: universal primer (Accurate Biology, Cat#: AG11716) |
| piR-62788 | F: TCCTGGGTTCGATTCCCAGC |
|  | R: universal primer (Accurate Biology, Cat#: AG11716) |
| U6 | F: Cat#: B661602 (Sangon) |
|  | R: universal primer (Accurate Biology, Cat#: AG11716) |
| Piwil1 | F: ATGATCGTGGGCATTGACTGTTACC |
|  | R: AACCTGGCATTGACTCGCTTCTTC |
| Piwil2 | F: GCCGCATCAGGAGATTGTGGAC |
|  | R: CAGGAGCAGCAAGGTACAGATTGG |
| Piwil4 | F: GCTTGGCGTCTCTTCTGGTGATG |
|  | R: GCCTTGTCGGAGAGTATGCTGTG |
| Srf | F: CCTCACCGTGCTCAATGCCTTC |
|  | R: GCGTCCAAGTTCACCACCTGTAG |
| Gapdh | Cat#: B661204 (Sangon) |

**Table S3. The sequences of oligonucleotides used in this study**

| **Oligonucleotides** | **Sequences** |
| --- | --- |
| piR-62788 mimic | uccuggguucgauucccagcauc  (monophosphate 5’ end, 3’ end 2’-OMe) |
| piR-62788 agomir | uccuggguucgauucccagcauc  (all the bases 2’-OMe, 3’ end cholesterol) |
| Locked nucleic acid (LNA) NC | IG mC mA IA mU mA IU mG mC IC mC mG IU mC mC IC mG mG IU mU mC IU mU  (I=LNA, m=2’-OMe) |
| piR-62788 LNA | IG mA mU IG mC mU IG mG mG IA mA mU IC mG mA IA mC mC IC mA mG IG mA  (I=LNA, m=2’-OMe) |
| *Piwil2* siRNA | UACUACCAGCUGCUCUUCC |

**Table S4. Baseline clinical and biochemical characteristics of the subjects**

|  | **Healthy controls**  **(n=13)** | **Patients with heart failure**  **(n=13)** | ***P* value** |
| --- | --- | --- | --- |
| **General variables** |  |  |  |
| Age, y | 56±6 | 58±11 | 0.659 |
| Sex, F/M (%/%) | 5/8 (38.5/61.5) | 6/7 (46.2/53.8) | 0.289 |
| BMI, Kg/m^2^ | 21.9±3.5 | 23.1±2.2 | 0.336 |
| **Risk factors** |  |  |  |
| Smoking, n (%) | 4 (30.8) | 6 (46.2) | 0.029 |
| Drinking, n (%) | 2 (15.4) | 4 (30.8) | 0.007 |
| **Medical variables** |  |  |  |
| Hypertension, n (%) | 0 | 4 (30.8) | – |
| CAD, n (%) | 0 | 3 (23.1) | – |
| VHD, n (%) | 0 | 3 (23.1) | – |
| DCM, n (%) | 0 | 5 (38.5) | – |
| **Medications** |  |  |  |
| RASI, n (%) | 0 | 6 (46.2) | – |
| β-blocker, n (%) | 0 | 4 (30.8) | – |
| CCB, n (%) | 0 | 2 (15.4) | – |
| Diuretics, n (%) | 0 | 7 (53.8) | – |
| **Cardiac variables** |  |  |  |
| SBP, mm Hg | 122±7 | 114±18 | 0.127 |
| DBP, mm Hg | 77±6 | 79±18 | 0.798 |
| Heart rate, bpm | 70 (57–76) | 95 (79–101) | <0.001 |
| **Biochemical variables** |  |  |  |
| ALT, U/L | 14 (11–20) | 22 (18–32) | 0.019 |
| Creatinine, μmol/L | 80 (63–85) | 93 (79–115) | 0.029 |
| TG, mmol/L | 1.02 (0.76–1.73) | 1.20 (0.77–1.83) | 0.448 |
| HDL-c, mmol/L | 1.29 (1.12–1.63) | 1.18 (0.98–1.30) | 0.243 |
| LDL-c, mmol/L | 3.45 (2.93–4.27) | 2.43 (2.13–2.74) | 0.001 |
| NT-proBNP, pg/ml | 39.1 (19.2–48.0) | 2923.0 (1682.5–7931.0) | <0.001 |
| **Echocardiography** |  |  |  |
| RVD, mm | 20.0 (17.0–23.5) | 25 (19.5–25.5) | 0.022 |
| LAD, mm | 31.3±3.8 | 43.9±6.7 | <0.001 |
| LVEDD, mm | 42.0 (40.0–46.5) | 67.0 (55.0–72.5) | <0.001 |
| LVESD, mm | 25.0 (22.5–28.5) | 58.0 (43.0–63.0) | <0.001 |
| LVEF, % | 67.4 (63.7–68.9) | 32.9 (27.0–38.2) | <0.001 |
| FS, % | 40.7±7.3 | 15.8±5.6 | <0.001 |

Values are given as mean±SD or as median (25th, 75th percentile) for continuous variables, and percentage for categorical data (n [%]). Abbreviations: BMI, body mass index; CAD, coronary artery disease; VHD, valvular heart disease; DCM, dilated cardiomyopathy; RASI, renin-angiotensin system inhibitor; CCB, calcium channel blocker; SBP, systolic blood pressure; DBP, diastolic blood pressure; ALT, alanine aminotransferase; TG, triglycerides; HDL-c, high density lipoprotein cholesterol; LDL-c, low density lipoprotein cholesterol; NT-proBNP, N-terminal pro-brain natriuretic peptide; RVD, right ventricular diameter; LAD, left atrial diameter; LVEDD, left ventricular end-diastolic diameter; LVESD, left ventricular end-systolic diameter; LVEF, left ventricular ejection fraction; FS, fraction shortening.
